# Analysis of the lipid body proteome of the oleaginous alga *Lobosphaera incisa*

**DOI:** 10.1186/s12870-017-1042-2

**Published:** 2017-06-06

**Authors:** Heike Siegler, Oliver Valerius, Till Ischebeck, Jennifer Popko, Nicolas J. Tourasse, Olivier Vallon, Inna Khozin-Goldberg, Gerhard H. Braus, Ivo Feussner

**Affiliations:** 1 University of Goettingen, Albrecht-von-Haller-Institute for Plant Sciences, Department of Plant Biochemistry, Goettingen, Germany; 2 University of Goettingen, Institute for Microbiology and Genetics, Department of Molecular Microbiology and Genetics, Goettingen, Germany; 3 UMR7141, CNRS/Université Pierre et Marie Curie, Paris, France; 4 Ben-Gurion University of the Negev, Microalgal Biotechnology Laboratory, Beer-Sheva, Israel; 5 University of Goettingen, Goettingen Center for Molecular Biosciences (GZMB), Goettingen, Germany; 6 University of Goettingen, International Center for Advanced Studies of Energy Conversion (ICASEC), Goettingen, Germany; 7 Present address: Laboratoire ARNA, INSERM U1212, CNRS UMR5320, Université Bordeaux 2; Institut Européen de Chimie et Biologie (IECB), 2 rue Robert Escarpit, 33607 Pessac, France

**Keywords:** Lipid bodies, Lipid droplets, *Lobosphaera incisa*, Nitrogen starvation, Oil bodies, Proteome, *Parietochloris*, TAG lipase

## Abstract

**Background:**

*Lobosphaera incisa* (*L. incisa*) is an oleaginous microalga that stores triacylglycerol (TAG) rich in arachidonic acid in lipid bodies (LBs). This organelle is gaining attention in algal research, since evidence is accumulating that proteins attached to its surface fulfill important functions in TAG storage and metabolism.

**Results:**

Here, the composition of the LB proteome in *L incisa* was investigated by comparing different cell fractions in a semiquantitative proteomics approach. After applying stringent filters to the proteomics data in order to remove contaminating proteins from the list of possible LB proteins (LBPs), heterologous expression of candidate proteins in tobacco pollen tubes, allowed us to confirm 3 true LBPs: A member of the algal Major Lipid Droplet Protein family, a small protein of unknown function and a putative lipase. In addition, a TAG lipase that belongs to the SUGAR DEPENDENT 1 family of TAG lipases known from oilseed plants was identified. Its activity was verified by functional complementation of an *Arabidopsis thaliana* mutant lacking the major seed TAG lipases.

**Conclusions:**

Here we describe 3 LBPs as well as a TAG lipase from the oleaginous microalga *L. incisa* and discuss their possible involvement in LB metabolism. This study highlights the importance of filtering LB proteome datasets and verifying the subcellular localization one by one, so that contaminating proteins can be recognized as such. Our dataset can serve as a valuable resource in the identification of additional LBPs, shedding more light on the intriguing roles of LBs in microalgae.

**Electronic supplementary material:**

The online version of this article (doi:10.1186/s12870-017-1042-2) contains supplementary material, which is available to authorized users.

## Background

Microalgae are a highly diverse group of organisms with regards to both their evolutionary and ecological background. Some species of microalgae accumulate the neutral lipid triacylglycerol (TAG) within the cells as a form of carbon and energy storage, a process that can be manipulated by exposure to different forms of abiotic stress. Among these, nitrogen starvation has been shown to result in increased TAG synthesis [1], a phenomenon that is accompanied by growth arrest [2–5], alterations in the proteome reducing the nitrogen content [6, 7], reduced photosynthesis on a transcription level [6, 8] and chloroplast degradation [3, 4, 6, 9, 10]. The fact that these changes are reversible makes oleaginous algae particularly interesting for studies of lipid metabolism.

TAG needs to be stored within the cell in a way that permits mobilization when needed. This function is fulfilled by cytosolic organelles the so-called lipid bodies (LBs) [11]. Apart from a core of neutral lipids, they consist of a monolayer of polar lipids and proteins that can be directly or indirectly attached to its surface [12]. A small number of structural elements is known that makes it possible for proteins to bind to LBs directly: A hydrophobic domain that protrudes into the LB [13], a “proline knot” [14] or “proline knob” [15] within this domain, a β-barrel [16] or amphipathic helices [17, 18]. However, a conserved amino acid sequence that targets proteins to the LB has so far not been identified.

In many organisms, LB proteins (LBPs) have been found to be involved in lipid metabolism or in maintaining LB structure [19, 20]. The former includes TAG lipases, which are required to remove the fatty acids from the backbone of the storage lipid. Mono- and Diacylglycerol (MAG and DAG) lipases may then cleave off the remaining two acyl moieties, which can subsequently be broken down by β-oxidation to access the stored energy. LB-associated TAG lipases have been described in yeast [21, 22], castor bean [23], rapeseed and mustard seed [24] as well as *Arabidopsis thaliana* (*A. thaliana*) seeds [25, 26]. Until recently, no TAG lipase activity has been established in any alga, with the exception of *Phaeodactylum tricornutum* TAG LIPASE1. It has been demonstrated that this enzyme is capable of hydrolyzing the TAG analog *para*-nitrophenyl butyrate in vitro and that suppressed expression of the gene leads to TAG accumulation in vivo [27], but a direct connection to TAG (rather than DAG or MAG) degradation has not been established to date.

The second category of LBPs comprises proteins that maintain the structural integrity of LBs. The most intensely studied family of structural LBPs was first described in maize seeds [28], has since been characterized in many species of higher plants and was named oleosins [29]. They have been accredited with functions in preventing LB coalescence, thus maintaining a high surface/volume ratio that is beneficial for rapid degradation when needed [30] and for freezing tolerance [31]. It has also been shown that oleosins need to be degraded in order for LB breakdown to take place [32]. Similar functions are served by perilipins in mammals [33, 34] and Drosophila [35] as well as LIPID DROPLET ASSOCIATED PROTEINs (LDAPs) in the vegetative tissue of olive, avocado and oil palm [36, 37]. Microalgae harbor yet another family of structural LBPs, the members of which are mostly called MAJOR LIPID DROPLET PROTEINs (MLDPs) and do not contain any of the known structural elements. The *C. reinhardtii* MLDP was the first one to be characterized [38] and it has since become evident that it recruits other proteins, particularly tubulins, to the LB surface [39]. Moreover, *C. reinhardtii MLDP* transcript abundance has been used as a marker for TAG accumulation [5]. Members of the MLDP family have been characterized in the microalgae *Haematococcus pluvialis* [10] and *Dunaliella salina* [3], while homologous genes can be found in the genomes of further members of the Volvocales and Chlorellales order [3]. The diatom *Phaeodactylum tricornutum* [40] and the heterokont microalga *Nannochloropsis* [41] each contain unique structural LB proteins, which, in contrast to MLDPs, contain prominent hydrophobic domains similar to oleosins.

*Lobosphaera incisa* is an oleaginous alga belonging to the class of Trebouxiophyceae. Here we investigated strain SAG 2468, which was originally isolated on a Japanese glacier [42]. It is unusual in accumulating large amounts of TAG that is rich in arachidonic acid (ARA, 20:4 (n-6)) [43]. Such partitioning of a PUFA into TAG is not common among microalgae [44] and nitrogen starvation can be used to further push the TAG content from 43% [43] to 87% of total fatty acids (TFAs), increasing the proportion of ARA at the same time [45].

The mitochondrial and plastidial genomes of this strain have been published [46, 47] and chemical mutagenesis has been successfully employed, resulting in a clear phenotype [48]. In addition, stable transformation has been achieved [49], albeit with low efficiency. A much larger array of molecular biology tools and resources is currently available for the model green alga *C. reinhardtii*, however this organism requires nutrient starvation to exhibit substantial LB formation. In contrast to this, the same process can be readily observed without manipulation in *L. incisa.*

So far, several algal LB proteomes have been analyzed, but only for very few postulated LBPs the subcellular localization has been verified by fluorescence microscopy [39, 41, 50] or immunolabelling [3]. In order to better understand lipid storage in the oleaginous alga *L. incisa*, the objective of this study was to identify proteins that associate with LBs and to verify this localization in order to reveal true LBPs. We furthermore used the recently sequenced nuclear genome as well as expression studies to reveal genes of interest for TAG degradation.

## Results

As LB-associated proteins have been found in many organisms, the *L. incisa* genome was initially searched for homologs of these. Neither oleosins, caleosins or steroleosins, which are known from oil seed plants, are encoded in the algal genome, nor could a homolog of plant LDAPs or mammalian perilipins be found. LBs were therefore isolated from *L. incisa* in order to identify novel LBPs.

### Putative LBPs were identified by a proteomics approach

**Fig. 1:**
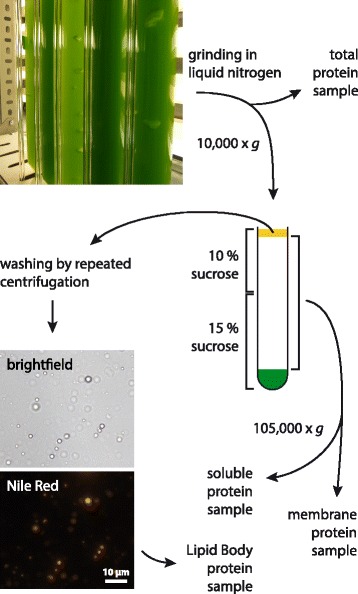
Overview of the cell fractionation procedure to obtain LBs and control samples from nitrogen-starved *L. incisa* culture

The steps required for LB isolation are outlined in Fig. 1. An *L. incisa* culture was starved of nitrogen for 3 days prior to cell fractionation, so as to promote LB formation. A large array of mechanical and enzymatic methods of cell disruption (as well as combinations thereof) had proven to be ineffective in breaking the adamant cell wall while leaving LBs intact, whereas grinding in liquid nitrogen yielded sufficient amounts of intact LBs as verified by Nile Red staining (Fig. 1). Repeated cycles of resuspension and centrifugation allowed partial removal of adhering membranes. Samples taken from the total extract, the soluble fraction and total membranes were used as controls in the subsequent identification of true LBPs.

The process of proteome data filtering leading up to this goal is summarized in Table 1. Proteins that had been detected in all three technical replicates of the LB sample were considered for further analysis, if they fulfilled at least one of four criteria: (i) Strong enrichment in the LB fraction compared to one or more control samples, (ii) high abundance in the LB fraction, (iii) a clear induction of gene expression in nitrogen-limiting conditions or (iv) homology with proteins that point to a function in lipid metabolism or LB homeostasis. Those candidates for which the coding sequences could be successfully amplified from cDNA were analyzed for subcellular localization. The genes of interest were fused to the reporter gene mVenus and transiently expressed in *Nicotiana tabacum* (*N. tabacum*) pollen tubes. This readily transformable tissue contains a large number of LBs and is therefore an ideal system to confirm the localization of putative LBPs of plant or algal origin [51].

**Table 1:** Filtering steps in the identification of possible LBPs based on high nanoLC-coupled mass spectrometry analysis

Filtering step	Number of proteins detected in LB samples		
None	484 ± 107		
Present in all 3 technical replicates	279		
	>10× enriched compared to any control sample or exclusively present in LB sample	AND/OR most abundant 10% in LB sample	AND/OR expression induced >5× under nitrogen limiting conditions
	134	28	23
Selected for further analysis, coding sequence successfully amplified by RT-PCR	11		
LB localization confirmed	3		

Only proteins were considered, for which at least two peptides had been detected with medium or high confidence. For the initial number prior to filtering, the mean of three technical replicates is given ± the standard deviation

The 11 proteins that were studied further are presented in more detail in Table 2. For most of the candidates investigated, at least ¼ of the amino acid sequence was covered by peptides detected (see Additional file 1). Enrichment in the LB fraction compared to the three control samples varies greatly, however out of the 5 most abundant proteins, three could be verified as LBPs and an additional protein was found to be partially associated with LBs.

**Table 2:** Proteins identified in *L. incisa* LB samples and selected for further analysis

	LB proteome					Transcription [FPKM]		LB localization
Protein name (accession)	Average NSAF ± SD [×10^−3^]	Coverage [%]	Fold enrichment compared to					
			Total protein extract	Membranes	Soluble protein	+N	3 d -N	
LiMLDP (g555.p1)	4.9 ± 0.1	75.5	1.0	1.6	0.9	261.0	901.4	yes
LiLBP62 (g15430.p1)	3.2 ± 1.4	64.8	31.3	*	26.6	47.8	62.3	yes
LiLBP36 (g13945.p1)	4.5 ± 1.0	49.1	5.5	7.3	9.2	151.6	1656.1	yes
g9582.p1	2.9 ± 0.4	39.0	21.9	*	76.8	17.7	25.9	partial
g9864.p1	4.0 ± 1.4	50.4	2.1	1.5	2.5	47.2	805.4	no
g13714.p1	1.7 ± 0.2	45.1	7.9	3.0	12.8	24.9	77.0	no
g4703.p1	1.5 ± 0.3	37.3	2.2	25.6	7.3	122.5	94.3	no
g13209.p1	1.3 ± 0.1	46.9	3.9	2.0	5.1	55.2	428.7	no
g12144.p1	1.2 ± 0.2	28.7	4.2	3.9	5.8	15.8	119.0	no
g13747.p1	1.2 ± 0.2	37.0	8.0	25.7	12.8	17.6	25.2	no
g14373.p1	0.7 ± 0.3	21.2	24.8	*	57.6	57.4	158.9	no

Only proteins that were detected in all three technical replicates were considered. Coverage refers to the proportion of the amino acid sequence covered by peptides detected in all samples. For each protein, fold enrichment in the LB sample compared to each control sample was calculated by division of the average Normalized Spectral Abundance Factors (NSAFs). Transcription levels in nitrogen replete conditions and after 3 d of nitrogen starvation were determined by RNA sequencing. LB localization was confirmed by heterologous expression in tobacco pollen tubes. SD, standard deviation of three technical replicates in a single experiment. FPKM, fragments per kilobase of transcript per million mapped reads. The asterisks denote samples, which did not contain the respective protein in all three technical replicates

### LiMLDP is a small and highly abundant LBP

**Fig. 2:**
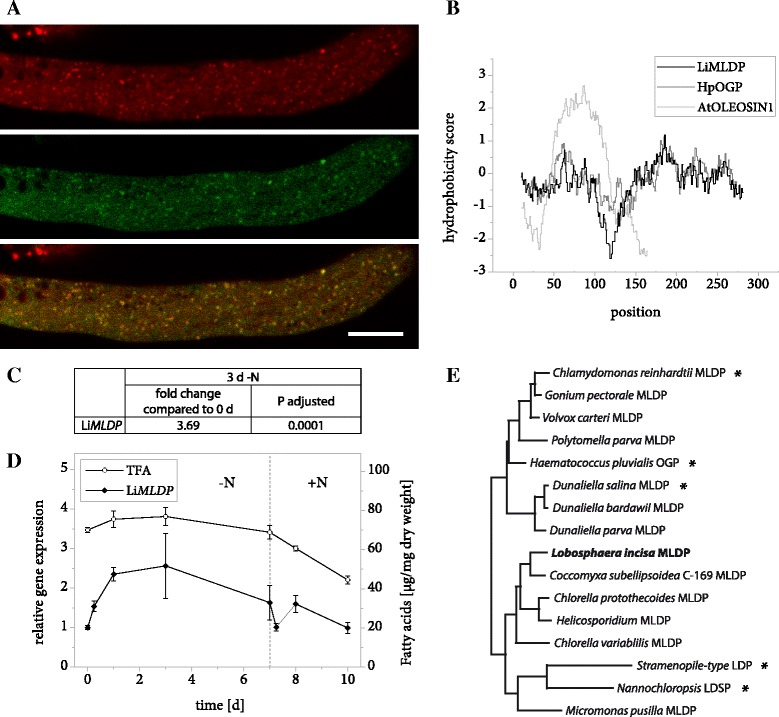
Properties of LiMLDP. **a** Subcellular localization of LiMLDP-mVenus in *N. tabacum* pollen tubes. 6 h after germination, cells were stained for LBs using Nile Red and fluorescence was documented by confocal laser scanning microscopy. From top to bottom: Nile Red, mVenus, merged image. Scale bar = 10 μm. 9 out of 9 pollen tubes analyzed showed comparable results. The characteristic punctate pattern of the mVenus signal was also observed in 10 out of 10 unstained pollen tubes. **b** Hydrophobicity of LiMLDP compared to HpOGP and *A. thaliana* OLEOSIN1 according to the Kyte & Doolittle amino acid scale. **c** Changes in LiMLDP expression in response to nitrogen starvation as determined by Illumina RNA sequencing. Samples from two *L. incisa* cultures were sequenced in 4 technical replicates each. The fold change is given after 3 days of nitrogen starvation compared to nitrogen replete conditions and the significance of this change is given as the *P* value adjusted for multiple testing at a false discovery rate of 0.05. **d** Changes in LiMLDP expression in response to varying nitrogen supply as determined by quantitative real-time PCR (qRT-PCR). Transcript levels were normalized to *RIBOSOMAL PROTEIN S21*. Expression is shown relative to time point 0 and error bars represent the standard error of the mean for three batches cultivated in parallel. The dotted line indicates the onset of nitrogen repletion and total fatty acid (TFA) levels are shown for comparison. **e** Phylogenetic tree of MLDPs determined by the maximum likelihood method. Proteins that have previously been studied are marked with an asterisk and LiMLDP is highlighted in bold. LDP = LIPID DROPLET PROTEIN, LDSP = LIPID DROPLET SURFACE PROTEIN

The protein encoded by gene *g555* was identified as an LBP candidate based on its high abundance in the LB protein extract (Table 2) and its similarity to a known algal LBP. A protein BLAST search revealed *Haematococcus pluvialis* oil globule protein (HpOGP) as the closest characterized homolog in any organism with 30% sequence identity, moreover the two proteins share a striking similarity in the distribution of hydrophobic residues along the amino acid sequence (Fig. 2b). These properties include the protein in the family of algal MLDPs (Fig. 2e) and the resemblance led us to name the *L. incisa* protein LiMLDP. Heterologous expression in *N. tabacum* pollen tubes confirmed its localization (Fig. 2a). Analyses of gene expression under conditions of nitrogen starvation and resupply indicate an upregulation during the first 3 days of growth limiting conditions (Fig. 2c and d, Additional file 2). The total fatty acid (TFA) content is shown for comparison (Fig. 2d, Additional file 3).

**Fig. 3:**
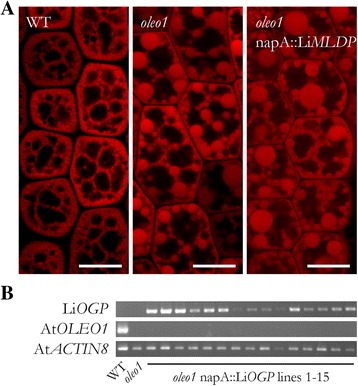
Heterologous expression of LiMLDP in the *A. thaliana oleo1* mutant background. **a** Confocal laser scanning microscope images of LBs in the hypocotyls of isolated embryos are shown. T2 seeds were preselected for expression of the *MCHERRY* reporter gene and imbibed at 4 °C over night before embryo isolation and LB visualization by Nile Red staining. For each of 15 independent lines, three embryos were observed. Shown in the right panel is a result for line 3, which is representative for all lines analyzed. **b** Gene expression of LiMLDP is shown for all 15 independent lines in the upper panel; the mutant background was verified in the middle panel. Gene expression of AtACTIN8 served as control in the lower panel

In contrast to oleosins, MLDPs including LiMLDP do not possess a prominent hydrophobic domain (Fig. 2b) that could directly anchor them at the LB surface. Nevertheless, LiMLDP appears to attach to LBs in some way and a possible function in maintaining LB structural integrity was tested. The gene was placed under the control of the seed specific *Brassica napus* napin A (napA) promoter and stably expressed in the *A. thaliana oleo1* mutant. LBs are dramatically enlarged in imbibed mutant seeds compared to the wildtype, however a functional complementation could not be observed in T2 seeds of 15 independent lines expressing LiMLDP (Fig. 3). See Additional file 4 for confirmation of gene expression.

### LiLBP62 localizes to LBs

**Fig. 4:**
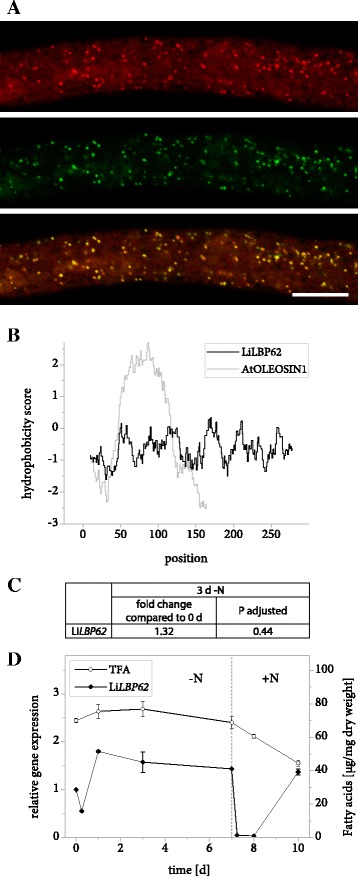
Properties of LiLBP62. **a** Subcellular localization of LiLBP62-mVenus in *N. tabacum* pollen tubes. Following 6 h of pollen germination, cells were stained for LBs using Nile Red and fluorescence was documented by laser scanning microscopy. From top to bottom: Nile Red fluorescence, mVenus fluorescence, merged image. Scale bar = 10 μm. 5 out of 5 pollen tubes analyzed showed comparable results. The characteristic punctate pattern of the mVenus signal was also observed in 10 out of 10 unstained pollen tubes. **b** Hydrophobicity of amino acid positions in the sequence of LiLBP62 compared to *A. thaliana* OLEOSIN1. The hydrophobicity score was determined using ExPASy ProtScale software with the Kyte & Doolittle amino acid scale and a window size of 19 residues. **c** Changes in expression of Li*LBP62* in response to varying nitrogen supply as determined by Illumina RNA sequencing. Samples from two *L. incisa* cultures were sequenced in 4 technical replicates each. The fold change of gene expression is given after 3 days of nitrogen starvation compared to nitrogen replete conditions and the significance of this change is given as the *P* value adjusted for multiple testing at a false discovery rate of 0.05. **d** Changes in expression of Li*LBP62* in response to varying nitrogen supply as determined by qRT-PCR. Transcript levels were normalized to *RIBOSOMAL PROTEIN S21*. Expression is shown relative to time point 0 and error bars represent the standard error of the mean for three batches cultivated in parallel. The dotted line indicates the onset of nitrogen resupply and total fatty acid (TFA) levels are shown for comparison

LiLBP62 is strongly enriched in the LB fraction of *L. incisa* compared to the control samples (Table 2) and also localizes to the same organelle in tobacco pollen tubes (Fig. 4a). Similarly to LiMLDP, it lacks a distinct hydrophobic domain (Fig. 4b). It contains a domain of unknown function (DUF 4057) [52, 53] that is absolutely specific of plants and green algae. Li*LBP62* undergoes no major expression changes over the 7-day course of nitrogen starvation, but the transcript drops down to below detection immediately following nitrogen resupply (Fig. 4c and d, Additional file 2).

Due to its high abundance at the LBs and its small size of 287 amino acids, a potential oleosin-like role for LiLBP62 in the structural integrity of LBs was investigated. Stable expression of the gene in the *A. thaliana oleo1* mutant as described above again did not reveal a complementation of LB size in imbibed T2 seeds of 13 independent lines.

### LiLBP36 is a putative LB lipase

**Fig. 5:**
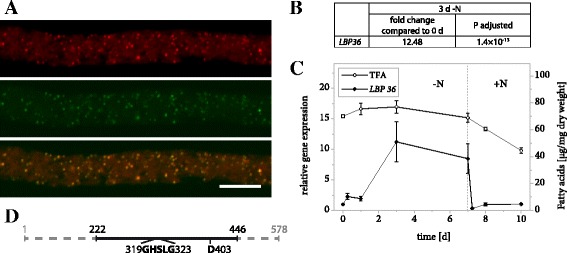
Properties of LiLBP36. **a** Subcellular localization of LiLBP36-mVenus in *N. tabacum* pollen tubes. Following 6 h of pollen germination, cells were stained for LBs using Nile Red and fluorescence was documented by laser scanning microscopy. From left to right: Nile Red fluorescence, mVenus fluorescence, merged image. Scale bar = 10 μm. 5 out of 5 pollen tubes analyzed showed comparable results. The characteristic punctate pattern of the mVenus signal was also observed in 13 out of 13 unstained pollen tubes. **b** Changes in expression of Li*LBP36* in response to varying nitrogen supply as determined by Illumina RNA sequencing. Samples from two *L. incisa* cultures were sequenced in 4 technical replicates each. The fold change of gene expression is given after 3 days of nitrogen starvation compared to nitrogen replete conditions and the significance of this change is given as the *P* value adjusted for multiple testing at a false discovery rate of 0.05. **c** Changes in expression of Li*LBP36* in response to varying nitrogen supply as determined by qRT-PCR. Transcript levels were normalized to RIBOSOMAL PROTEIN S21 transcripts. Expression is shown relative to time point 0 and error bars represent the standard error of the mean for 3 batches cultivated in parallel. The dotted line indicates the onset of nitrogen repletion and total fatty acid (TFA) levels are shown for comparison. **d** Schematic representation of LiLBP36 features with numbers indicating amino acid positions. The full line illustrates the section of the amino acid sequence that bears strong resemblance to fungal TAG-, DAG- and MAG- lipases according to secondary structure modelling using Phyre2. The section includes the conserved GXSXG motif as well as a second catalytic residue (D403)

LiLBP36 is not only highly abundant at *L. incisa* LBs, but also at least 5× enriched compared to all three control samples (Table 2) and the subcellular localization could be clearly confirmed in tobacco pollen tubes (Fig. 5a). The corresponding transcript levels respond to nitrogen starvation with a sharp increase within 3 days and drop to their initial value upon nitrogen resupply (Fig. 5b and c, Additional file 2).

LiLBP36 was annotated by Interproscan as a class 3 lipase, and the predicted secondary structures of a section of 224 amino acids (see Additional file 5) match very closely the experimentally determined structures of a group of fungal and yeast secreted lipases that act on MAG, DAG and TAG. These enzymes contain a catalytic triad of serine, aspartate and histidine that are found in many lipases [54], the serine residue being part of a conserved GXSXG motif. Except for the histidine residue, all of these elements are present in the structurally homologous section of LiLBP36 (Fig. 5d).

**Fig. 6:**
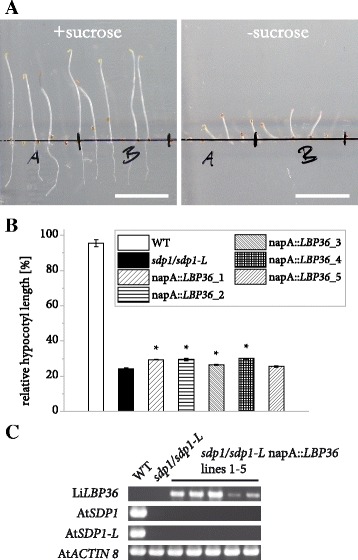
Impact of heterologous Li*LBP36* expression on postgerminative growth in *A. thaliana sdp1/sdp1-L* seedlings. **a** Elongation of *sdp1/sdp1-L* napA::*LBP36* line 2 seedlings after 5 d in the dark with or without sucrose. Bars = 1 cm. **b** Quantification of hypocotyl length for 5 independent lines as well as WT and mutant lines carrying an empty vector. For both growth conditions and each independent line, three or 4 batches of at least 15 seedlings were measured and hypocotyl length of seedlings germinated without sucrose was divided by the average value for the same line with sucrose. Error bars represent the standard error of the mean. Asterisks denote measurements on transgenic lines deviating significantly from the mutant (two-sided Student’s T-test, α = 0.05). **c** Confirmation of gene expression. Transcripts were detected in dry seeds of independent lines with the *Brassica napus *napin A (napA) promoter controlling expression of *L. incisa* *sdp1/sdp1-L*

Based on these observations along with the strong association with LBs, we suspected a TAG lipase activity for this protein. We therefore investigated the ability of LiLBP to complement TAG degradation in an *A. thaliana* mutant lacking the major TAG lipases that drive postgerminative growth: SUGAR-DEPENDENT1 (SDP1) and SDP1-LIKE [26]. Etiolated *sdp1/sdp1-L* seedlings require sucrose in order to form equally long hypocotyls as the wildtype, a property that permits an indirect observation of TAG breakdown [25, 26]. We introduced the Li*LBP36* gene into the mutant under the control of the napA promoter and measured the hypocotyl length of T2 seedlings after 5 days of germination in the dark in the presence or absence of sucrose in the media (Fig. 6a). For each independent line, the average hypocotyl length with sucrose was then set to 100% the relative length that was reached without sucrose was calculated and then compared to mutant and wildtype seedlings carrying only an empty vector (Fig. 6b). In 4 out of 5 lines, a modest improvement of postgerminative growth could be observed, however a strong functional complementation could not be ascertained (Fig. 6b and c, Additional file 6). See Additional file 4 for confirmation of gene expression.

### Several LBP candidates do not localize to LBs when tested in *N. tabacum* pollen tubes

The search for LBPs yielded 8 more candidates (see Table 2) that were further analyzed. g9582.p1 was selected based on its strong enrichment in the LB fraction and its Interproscan annotation as a class 3 lipase. When transiently expressed in tobacco pollen tubes, the protein localized to some LBs but was also abundant in other parts of the cell. *g9864* expression is drastically upregulated in response to nitrogen starvation and encodes a protein of unknown function that accumulates to some extent at the LBs. g13714.p1, a protein with high similarity to *A. thaliana* fatty acid amide hydrolase, is clearly enriched at LBs. At this point, a possible function for g4703.p1 cannot be predicted, but Interproscan reveals a sterile alpha motif possibly involved in protein binding and the protein is much more abundant in the LB fraction than in total membranes. *g13209* encodes a predicted short chain dehydrogenase/reductase and is highly expressed during nitrogen starvation. The putative FAD-binding monooxygenase g12144.p1 is similarly induced, while g13747.p1 and the patatin-related g14373.p1 are characterized by a striking accumulation at the LBs compared to the control samples. These 7 candidates did not localize to the LBs when expressed in tobacco pollen tubes and were thus not investigated further in this study.

### LiSDP1 is a TAG lipase

**Fig. 7:**
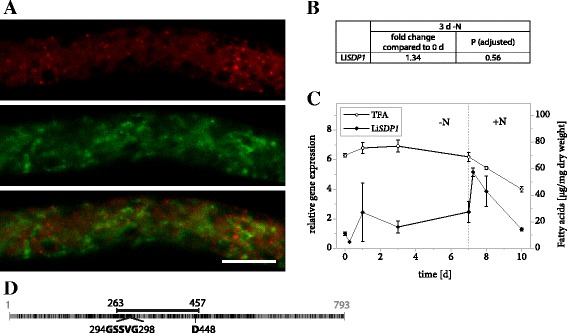
Properties of LiSDP1. **a** Subcellular localization of LiSDP1-mVenus in *N. tabacum* pollen tubes. Following 6 h of pollen germination, cells were stained for LBs using Nile Red and fluorescence was documented by laser scanning microscopy. From left to right: Nile Red fluorescence, mVenus fluorescence, merged image. Scale bar = 10 μm. 8 out of 8 pollen tubes analyzed showed comparable results. **b** Changes in expression of Li*SDP1* in response to varying nitrogen supply as determined by Illumina RNA sequencing. Samples from 2 *L. incisa* cultures were sequenced in 4 technical replicates each. The fold change of gene expression is given after 3 days of nitrogen starvation compared to nitrogen replete conditions and the significance of this change is given as the *P* value adjusted for multiple testing at a false discovery rate of 0.05. **c** Changes in expression of Li*SDP1* in response to varying nitrogen supply as determined by qRT-PCR. Transcript levels were normalized to RIBOSOMAL PROTEIN S21 transcripts. Expression is shown relative to time point 0 and error bars represent the standard error of the mean for 3 batches cultivated in parallel. The dotted line indicates the onset of nitrogen repletion and total fatty acid (TFA) levels are shown for comparison. **d** Schematic representation of LiSDP1 features with numbers indicating amino acid positions. Black segments represent residues that are identical or similar to *A. thaliana* SDP1 and the full line illustrates the conserved patatin domain. It includes the conserved GXSXG motif as well as the catalytic residue D448

We were surprised not to find a TAG lipase among the proteins that passed our criteria for filtering LB proteomics data and therefore turned to the *L. incisa* genome in search of promising candidates. This identified a protein that shares 44% identity with *A. thaliana* SDP1 and that was therefore named LiSDP1. Like its plant homolog, the protein features a patatin domain that is characteristic of a diverse group of lipases [55]. The domain harbors a catalytic dyad consisting of a serine residue within a conserved GXSXG motif as well as an aspartate residue (Fig. 7d). That LiSDP1 was not a LB protein was confirmed by transient expression in *N. tabacum* pollen tubes, which showed no overlap of the LiSDP1-mVenus and Nile Red signals (Fig. 7a). Gene expression only underwent minor changes during nitrogen starvation (Fig. 7b and c, Additional file 2), whereas it markedly increased within 6 h of nitrogen resupply, a growth phase characterized by substantial fatty acid mobilization (Fig. 7c).

**Fig. 8:**
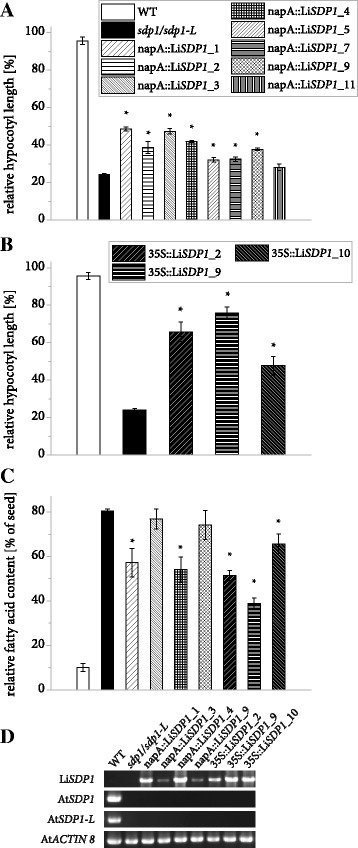
Functional complementation of postgerminative growth in etiolated *A. thaliana sdp1/sdp1-L* seedlings by Li*SDP1* expression. **a** Quantification of hypocotyl length for WT, mutant and complemented lines after 5 d of germination in the dark with or without sucrose. Transgene expression was under the control of the *Brassica napus* napinA (napA) promoter. For both growth conditions and each independent line, 3 or 4 batches of at least 15 seedlings were measured and hypocotyl length of seedlings germinated without sucrose was divided by the average value for the same line with sucrose. Error bars represent the standard error of the mean. Asterisks denote measurements on transgenic lines deviating significantly from the mutant (two-sided Student’s T-test, α = 0.05). **b** Effect on the hypocotyl length of Cauliflower Mosaic Virus 35S (35S) promoter controlling expression of the Li*SDP1* gene. **c** Relative fatty acid content of etiolated seedlings compared to seeds. Total fatty acids were derivatized by acidic methanolysis and analyzed by gas chromatography. For each independent line, three or 4 batches of 10 seeds and three or 4 batches of at least 9 seedlings were measured and the seedling fatty acid content was divided by the average value for seeds of the same line. Error bars represent the standard error of the mean. Asterisks denote measurements on transgenic lines deviating significantly from the mutant (two-sided Student’s T-test, α = 0.05). **d** The gene expression of LiSDP1 driven by the two promoters is shown in the first panel. In addition, the mutant background was verified in the two middle panels, whereas the gene expression of ACTIN8 was used as a control in the lowest panel

TAG lipase activity was tested by complementing the *A. thaliana sdp1/sdp1-L* mutant as described above. In 7 out of 8 independent lines carrying the Li*SDP1* gene controlled by the napA promoter, a significantly improved postgerminative growth could be observed compared to the mutant (Fig. 8a) and three lines expressing the gene constitutively showed an even stronger effect (Fig. 8b, see Additional file 4 for confirmation of gene expression). The assay was extended for selected lines by quantifying fatty acids in seedlings and relating them to the average seed fatty acid content of the same line (Fig. 8c, Additional file 7). In wildtype seedlings, the majority of fatty acids is metabolized during postgerminative growth, whereas the mutant retains 80% of fatty acids. For most complemented lines, the analysis revealed a significantly reduced amount of residual fatty acids after 5 d of germination without light or sucrose.

## Discussion

LBs are not considered as passive cell components anymore and interest in the functions they harbor is growing [11]. There is a high degree of variation in the amount and identity of LBPs that have been reported in previous studies even within the same algal species. This becomes obvious when comparing recent LB proteome studies in *C. reinhardtii*: Different strains were all cultivated photoheterotrophically and while one study did not reveal a single LBP [56], other groups reported 259 [38] and 248 [57], with an overlap of less than half. Co-immunoprecipitation with *C. reinhardtii* MLDP revealed 124 additional proteins that had not been found by the same group in the same strain [39]. This high degree of variation may illustrate the strong influence of sample preparation on the detection of LBPs in proteomics studies.

### Filtering of proteomics data revealed true LBPs

In this study, highly sensitive liquid chromatography-tandem mass spectrometry (LC-MS/MS) yielded a large number of putative LBPs. LB isolates from microalgae often contain large amounts of proteins originating from other cellular components [57–60], which can be due to an interaction of other organelle membranes with the LB surface [61] or contamination as a result of the cell fractionation process. This explains why extensive filtering of proteomics data was necessary in order to identify the three clearly LB-associated proteins described in this study.

A contamination of the LB fraction with plastoglobules could also be feared, but this seems unlikely. To date, the plastoglobule proteome has been characterized in *A. thaliana* and the green alga *Dunaliella bardawil* [62, 63] and for both organisms, only very little overlap with the LB proteome was found. In an electron microscopy study of *L. incisa*, no plastoglobules could be observed [64] and like other chlorophytes, the *L. incisa* genome lacks a homolog of plastoglobulin, which is characteristic of these structures [65].

The proteins that have been identified as LBPs in other algae so far can be grouped into highly abundant structural proteins [3, 10, 38, 40, 41, 66, 67] and enzymes that play a role in lipid metabolism or trafficking [38, 57]. Confirmation of the subcellular localization is essential in investigating the LB proteome and tobacco pollen tubes are a useful tool to do this [51]. In our study, three proteins could be identified as true LBPs.

While oleosins in higher plants are anchored in the LB surface via a hydrophobic domain that contains a proline knot [13], no such element can be found in any of the LBPs described here. Mammalian perilipins attach by means of amphipathic helices, which are formed by tandem repeats in the amino acid sequence [18]. A search for such repeats in the LiLBPs described here using HHrepID [68, 69] yielded no results. This is also the case for a range of other proteins that are known to associate with LBs including algal MLDPs [25, 38, 70]. These proteins possibly attach to the LB surface through acylation or by interaction with other proteins.

### LiMLDP and LBP62 are LB-associated proteins of unknown function

LiMLDP has similarities to other algal MLDPs: some sequence homology, a highly comparable distribution of hydrophobicity along the amino acid sequence and a clear induction of gene expression in early stages of nitrogen starvation. Unlike *Nannochloropsis* LDSP (lipid droplet surface protein) [41] however, in our experiment LiMLDP is unable to complement the high surface/volume ratio of LBs in *A. thaliana oleo1* embryos. This may be due to the napA promoter driving gene expression at too low a level or at an inappropriate time during seed maturation. A different role for this protein is also conceivable, for instance in recruitment of enzymes to the LB. Similar functions can be proposed for LiLBP62, which bears no features that could point to a particular physiological role. It is likely to be involved in LB formation or homeostasis rather than LB breakdown, since transcription of the Li*LBP62* gene rapidly ceases within 6 h of nitrogen resupply.

### LBP36 is a LB-localized putative lipase

In spite of its showing only weak sequence similarity to other known protein, LiLBP36 can be classified as a putative lipase based on predicted structural resemblance to a group of yeast and fungal lipases degrading MAG, DAG and TAG. This group of enzymes includes the first TAG lipase for which a crystal structure was solved and a catalytic triad of serine, histidine and aspartate identified in the active site [71]. Modeling LiLBP36 on the experimentally determined structure of this enzyme permits the identification of catalytic residues, except for the catalytic histidine, which is located in a section of the crystal structure that does not exhibit extensive homology with the modeled LiLBP36 structure.

Expression of the LiLBP36 gene appears to contradict a possible involvement in TAG degradation, as transcription is strongly induced during nitrogen starvation, whereas it decreases as soon as nitrogen is resupplied and lipid reserves are degraded. Nevertheless, gene expression could be induced in anticipation of a future need for TAG hydrolysis, possibly resulting in an inactive form of the protein that can be activated as soon as environmental conditions permit growth once again. A similar reasoning has been proposed to explain the expression pattern of the major TAG lipase in *A. thaliana* seeds, SDP1 [25].

In spite of these similarities with TAG lipases, LiLBP36 only effected a minor complementation of postgerminative growth in etiolated *A. thaliana sdp1/sdp1-L* seedlings. This could be due to a slow translation rate for this protein caused by differences in codon usage, considering that the LiLBP36 coding sequence contains almost 62% guanidine and cytosine residues, while the average for *A. thaliana* coding sequences is 44% (The Arabidopsis Information Resource, TAIR). Another possible explanation lies in the substrate specificity, as it is extremely difficult to predict the exact substrate range solely based on the modeled structure of a lipase. The distribution of molecular TAG species in *L. incisa* (mostly 18:1 (n-9) and ARA, [72]) differs from that in *A. thaliana* seeds (mostly 18:1 (n-9), 18:2 (n-6), 18:3 (n-3) and 20:1 (n-9), [73]) and a TAG lipase that acts on one TAG pool will not necessarily act with high efficiency on the other. Among neutral lipids, the enzyme may have a higher affinity for MAG or DAG, neither of which were tested in this study. Alternatively, it may have a primary function in hydrolyzing membrane lipids that form the polar monolayer on the LB surface, thereby permitting subsequent TAG degradation by other lipases.

### LiSDP1 is a TAG lipase

The proteomics approach employed in this study proved useful in the identification of LBPs and the LB proteome dataset can serve as a resource for the identification of additional LBPs. We were surprised to find that the *L. incisa* homolog of SDP1 is not present in our LB protein dataset. We were interested in TAG hydrolysis and therefore went on to analyze this gene in more detail. SDP1 and its paralog SDP1-LIKE have been shown to degrade TAG in *A. thaliana* [25, 26, 74].

Our analysis of Li*SDP1* transcript levels revealed that gene expression is rapidly induced as soon as nitrogen is no longer limiting in the culture and lipid mobilization begins. We observed that the enzyme is in fact able to take over a TAG lipase function when introduced into the *A. thaliana sdp1/sdp1-L* mutant. The fatty acid profile of seedlings and seeds did not differ significantly between WT and transgenic lines, pointing to a broad substrate specificity of *L. incisa* SDP1 as reported for its *A. thaliana* homolog [25]. The overall level of TAG lipase activity exerted by the algal enzyme appeared low, possibly owing to a specialization for TAG species that are absent from *A. thaliana* seeds. The effect on postgerminative growth of etiolated seedlings was most pronounced when expression was controlled by the 35S promoter, which has been shown to be equally effective as the endogenous *A. thaliana* sdp1 promoter in complementing the *sdp1* mutant [26]. The napA promoter effected a weaker complementation, which may be due to a lower level or an unfavorable temporal course of gene expression, even though the promoter is reportedly active not only during seed maturation but also during germination in *Brassica oleracea* [75].

The observations that LiSDP1 was not present in our LB isolate from nitrogen starved algae and that it does not localize to the LBs in tobacco pollen tubes suggests that the TAG lipase may be among the proteins that are specifically recruited to the LBs in *L. incisa* when needed. It has been shown for *C. reinhardtii* that the LB proteome is subject to vast changes in response to nitrogen resupply [39]. However, recent evidence obtained for *A. thaliana* seedlings may support our findings. Here the picture emerges that SDP1 may be rather localized at the peroxisomal membrane and is only translocated to the LB membrane upon TAG mobilization [76, 77]. The gene expression pattern may support this notion, as LiSDP1 expression is only induced in response to nitrogen resupply.

## Conclusions

*L. incisa* is an oleaginous microalga that holds great promise for the study of lipid metabolism. In this study, an extensive LB proteome dataset was generated and successfully filtered for true LBPs. *N. tabacum* pollen tubes were used as an efficient tool for the verification of LB localization in our experimental setup. Two LBPs of unknown function are suspected to play a role in maintaining the structural integrity of the organelle and preventing its premature degradation, while another is a promising candidate for lipase activity that could be required for storage lipid mobilization. Failure to confirm the LB localization of other candidate LBPs that were investigated here emphasizes the necessity of verifying results from cell fractionation. At the same time, the use of a heterologous system such as tobacco pollen possibly entails differences in targeting signal recognition or a lack of certain scaffolding proteins required for LB localization. Nevertheless, the dataset generated here will be a useful resource for the identification of further LBPs, possibly revealing novel aspects of LB metabolism and homeostasis.

Screening the *L. incisa* genome for further lipase candidates revealed a homolog of the major TAG lipase in *A. thaliana* seedlings, Li*SDP1*. The algal gene was able to reconstitute hypocotyl elongation in etiolated seedlings lacking endogenous SDP1 and SDP1-L, verifying TAG lipase activity for this enzyme. The enzyme does not localize to the LBs in nitrogen-starved *L. incisa* or the tobacco pollen tube system, instead it is likely to be recruited to the sites of TAG storage when these reserves need to be mobilized. The robust cell wall of *L. incisa* is a major obstacle in the isolation of relatively pure organelles.

## Methods

### *L. incisa* Genome sequencing

Assembly of the nuclear genome of *L. incisa* was carried out along with that of the mitochondrial and plastidial genomes as described previously [46]. Briefly, Illumina paired-end and Long Jumping Distance mate pair reads were assembled using the super-read-based assembler MSR-CA (MaSuRCA) [78]. Protein-coding genes were predicted with AUGUSTUS 2.6.1 (PMID:16469098) [79] and annotated using InterProScan 5.9 (PMID:24451626) [80], USEARCH/UBLAST 7.0.1001 (Edgar 2010, Bioinformatics 26, 2460–2461) and KEGG (using the KAAS server: Moriya et al. 2007, Nucleic Acids Research, 35, W182–W185). The sequences and annotations are available at https://giavap-genomes.ibpc.fr. Please contact ovallon@ibpc.fr for authorizations.

### Liquid culture of *L. incisa*

*L. incisa* strain SAG 2468 had originally been isolated on a Japanese glacier [42] and was kindly provided by Dr. Inna Khozin-Goldberg, Ben-Gurion University of the Negev, Israel. It was cultivated in BG11 media [81] in 300 mL volumes at 25 °C and subject to continuous illumination with 190 μmol photons m^−2^s^−1^ as well as aeration with a supplement of 1% (*v*/v) CO_2_. Nitrogen starvation was achieved by washing and resuspending the cells in BG11 media which was modified by omission of NaNO_3_ and replacement of ammonium ferric citrate with ferric citrate [45]. Nitrogen was resupplied by sedimenting cells and once again taking them up in full BG11 media.

### Gas chromatography

Fatty acids in algal material were quantified by GC as previously described [82] with minor modifications. Following lyophilization of the material, lipids were isolated by Methyl *tert*-butyl ether (MTBE) extraction according to [83] and fatty acids were derivatized by acidic methanolysis [84]. The temperature gradient employed in GC measurements comprised the following steps: 150 °C for 1 min, 150 °C to 200 °C at 4 °C/min, 200 °C to 250 °C at 20 °C/min and 250 °C for 3 min.

*A. thaliana* seeds were dried at 60 °C over night and frozen seedlings (including seed coats) were lyophilized prior to direct acidic methanolysis [84]. Tri-15:0 was included as an internal standard for quantification and samples were mechanically disrupted during the reaction using a metal rod to ensure efficient methanolysis.

### LB isolation

Centrifugation steps were generally carried out at 4 °C in an Eppendorf 5810R centrifuge, an Optima LE-80 K ultracentrifuge with an SW40 rotor or an Optima TLX ultracentrifuge with a TLS55 rotor for 50 mL tubes, 12 mL thin wall polypropylene tubes and 2.5 mL thin wall polypropylene tubes, respectively. Ice-cold buffers were used and samples were kept on ice between fractionation steps.

An *L. incisa* culture was starved of nitrogen for 3 days prior to LB isolation. Cells from 50 mL of culture were sedimented by centrifugation at 2500 x *g* for 10 min, washed once with distilled water and then ground in liquid nitrogen. LBs were subsequently isolated according to [85] with some modifications. 60 mL centrifugation buffer A (100 mM Tris-HCl pH 7.5, 3 mM ethylenediaminetetraacetic acid (EDTA), 10 mM Dithiotreitol (DTT), 1% (*v*/v) Plant Protease Inhibitor Cocktail (Sigma-Aldrich), 0.6 M sucrose) were added to the homogenized material and a 200 μL sample of the total cell extract was taken before removal of cell debris by centrifugation at 10,000 x *g* for 10 min. The entire supernatant was transferred to 12 mL tubes, each was carefully overlain with 10 mL centrifugation buffer B (buffer A with 0.4 M sucrose) and centrifugation was repeated. LBs floating on top were removed with a spatula and the remaining supernatant was separated into membranes and a soluble fraction by ultracentrifugation at 105,000 x *g* for 90 min. A 200 μL sample of the soluble fraction was taken and the sedimented membranes were combined. The LBs were transferred to a Potter-Elvehjem tissue grinder and carefully resuspended in 10 mL centrifugation buffer A. Overlaying with 8 mL buffer B and another centrifugation step at 10,000 x *g* yielded an LB fraction floating on top, which was then washed this way two more times. Finally, LBs were resuspended in 1.5 mL buffer A, transferred to a 2.5 mL ultracentrifugation tube and overlain with buffer B before ultracentrifugation at 100,000 x *g* for 1 h.

### LB protein identification

Proteins were extracted from each cell fraction without further separation as described previously [85, 86] with some modifications. They were precipitated in 90% ethanol at −80 °C for 2 h and sedimented at 20,000 x *g* and 4 °C for 15 min followed by washing with 80% ethanol three times. Proteins were solubilized in 100 μL denaturing protein solubilization buffer (4% sodium dodecyl sulfate (SDS), 4 mM DTT, 8% (*v*/v), 80 mM Tris-HCl pH 6.8, 0.02% (*w*/*v*) Bromophenolblue, 7 M urea, 2 M thiourea [59, 87] at 37 °C for 2 h before applying each sample in triplicate to SDS-PAGE [87–90] until it had migrated 1 cm. The gel was stained with Coomassie [91, 92], destained in water and the entire band was excised. The proteins were then subjected to an in-gel tryptic digest as described previously [93] and peptides were identified by liquid chromatography coupled to tandem mass spectrometry (see Additional file 1 for details). Proteins were then identified by comparison to a list of all *L. incisa* proteins that had been predicted based on the nuclear and plastidial genomes.

Proteins that were detected in all three technical replicates of the LB sample were considered for further analysis and for each one, abundance was estimated by calculating the Normalized Spectral Abundance Factor (NSAF, [94, 95]). Enrichment compared to the three control samples (total extract, membranes, soluble fraction) was determined by dividing NSAFs.

### Protein in silico analyses

Homologous proteins and conserved domains were searched using BLASTP [96, 97] and the PFAM database [52, 53], respectively.

Phyre2 [98, 99] was used to predict protein secondary structures and to search for structural homologs.

Hydrophobicity of amino acid sequences was determined using ExPASy ProtScale [100, 101] with the Kyte & Doolittle amino acid scale [102] and a window size of 19 residues.

The phylogeny of related proteins was inferred using the maximum likelihood method with PhyML [103, 104] following sequence alignment with Clustal Omega [105, 106].

### Coding sequence amplification and plasmid construction

RNA was isolated from an *L. incisa* culture that had been deprived of nitrogen for 3 days and cDNA was synthesized (see below). Coding sequences of interest were amplified and restriction sites were added using the primers listed in Additional file 8 before ligation into a subcloning vector using the CloneJET PCR cloning kit (Thermo Fisher Scientific).

The coding sequences without a stop codon were inserted into the pUC-LAT52-mVenus vector [107, 108] for expression in tobacco pollen tubes by restriction cloning using endonucleases obtained from Thermo Fisher Scientific. Similarly, the full coding sequences were inserted into the pEntry-E vector [109] for further transfer into pCambia plant expression vector 43.0 by Gateway cloning technology. pCambia 43.0 had been as a new expression vector with mCherry as a reporter gene in plants under the control of the seed specific napin promoter from *Brassica napus*. To generate the vector, a napin::mCherry construct was amplified via PCR, creating restriction sites SacI and XhoI at the 5′ and 3′-end. pCambia33.0G was digested with SacI and XhoI and ligated with the napin::mCherry construct to substitute for the *bar* gene under the control of the 35S promoter. For vector details see Additional file 9.

### Transient gene expression in tobacco pollen tubes

Transient expression in tobacco pollen tubes was used to verify the LB localization of LBP candidates as previously described [51]. *N. tabacum* (ecotype Samsun NN) pollen grains were transiently transformed by particle bombardment as described previously and allowed to germinate on glass slides for 6 h. Pollen tubes were then fixed with PT media [110] containing formaldehyde (1% (*w*/*v*) final concentration) and stained for LBs with Nile Red (0.17 ng/μL final concentration).

### Confocal microscopy

Fluorescence images were obtained using a Zeiss LSM Meta confocal microscope. Nile Red and mVenus were excited at 561 and 488 nm, imaged with HFT 405/488/561 and HFT 405/514/633 nm major beam splitters and fluorescence was detected at 571-593 and 507-539 nm, respectively.

### Stable transformation of *A. thaliana*

The *A. thaliana* mutants *sdp1/sdp1-L* [26]) and *oleo1* (SM_3.29875, kindly provided along with the corresponding wildtype by Prof. Dr. Christoph Benning, Michigan State University) were transformed with pCambia vectors containing algal genes along with the *MCHERRY* reporter gene by the floral dip method using *Agrobacterium tumefaciens* strain EHA 105 [111]. The mutants as well as the Col-0 wildtype were also transformed with an empty vector to generate lines to be used as negative controls. T1 and T2 seeds expressing the reporter gene were identified by fluorescence microscopy using an M165C stereomicroscope equipped with an EL6000 mercury lamp, a M205FA/M165FC filter set and a DFC3000 G camera (all from Leica Microsystems). Gene expression was confirmed as described below.

### Gene expression analyses

For RNAseq, *L. incisa* cells grown in control conditions or nitrogen-starved for 12 or 72 h, either in low light (75 μmol photons.m^−2^.s^−1^) or in high light (150 μmol photons.m^−2^.s^−1^), from two biological replicates. RNA was isolated from frozen samples using the SV Total RNA isolation kit (Promega) after breaking the material with iron beads in liquid nitrogen. RNA quality was examined on a 2100 Electrophoresis Bioanalyzer. The transcriptome was sequenced using the Illumina Truseq high-throughput short-read technology (using 50-nt single-end and 100-nt paired-end reads) on a HiSeq 1000 instrument by the transcriptomics platform of the Institut de Biologie de l’École Normale Supérieure (Paris, France). For each growth condition, two biological replicate samples were sequenced, each in four technical replicates. They were mapped onto the transcripts and analyzed for differential expression using the protocol of Haas et al. [112] using tools from the Trinity (release r20131110), RSEM 1.2.9 [113], and DESeq [114], the latter also providing an analysis of deviance (ANODEV). A false discovery rate of 5% was used for cutoff.

For selected genes, RNA-Seq results were confirmed and expanded by means of qRT-PCR. For this purpose, total RNA was isolated from three *L. incisa* cultures at various time points during nitrogen starvation as well as following nitrogen resupply. Samples were freeze-dried and subsequently ground in liquid nitrogen to ensure efficient cell disruption prior to Trizol extraction [115]. DNase I and RevertAid H Minus (Thermo Fisher Scientific) were then used to remove residual genomic DNA and to synthesize cDNA. Primer3Prefold [116] and Primer3Plus [117] were used for primer design (see Additional file 8 for primer sequences) and *RIBOSOMAL PROTEIN S21* was used as a reference gene for normalization. qRT-PCR was carried out with an iQ5 qPCR cycler (Biorad Laboratories) and the Takyon No Rox SYBR Core Kit blue dTTP (Eurogentec).

In dry seeds of *A. thaliana*, gene expression was confirmed by extracting RNA as previously described [118], generating cDNA as outlined above and detecting transcripts by PCR.

### Postgerminative growth assay

Hypocotyl length was used to evaluate postgerminative growth in etiolated seedlings of *A. thaliana sdp1/sdp1-L* mutant lines expressing putative TAG lipase genes as previously described [26]. Seed-specific or constitutive transgene expression were controlled by the *Brassica napus* napin A promoter or the Cauliflower Mosaic Virus 35S promoter, respectively. T2 seeds were preselected for a size of 250 – 300 μm, surface sterilized and lined up on ½ MS agar plates with or without 1% sucrose. Reporter gene expression was documented by fluorescence microscopy and germination was synchronized by stratification at 4 °C for 2 days. Seeds were then exposed to light for 30 min to induce germination and afterwards kept at room temperature in the dark for 5 days. At this point seedlings were documented using a scanner and for those that originated from transgenic seeds, hypocotyl length was measured. For each line, 4 batches of at least 10 seedlings were measured. Gas chromatography was used to determine the TFA content for these seedlings (including seed coats) as well as transgenic seeds of the same line, using the configurations as detailed above. For each line, 4 batches of at least 10 seedlings were analyzed and the TFA content in each batch was divided by the number of seedlings. The 4 values for TFA per seedling were then used to calculate the mean value and standard error of the mean for each line. Transgenic seeds were analyzed in the same way.

### LB size assay

The impact of algal proteins on the structural integrity of LBs was investigated by expressing the corresponding genes in the *A. thaliana* mutant *oleo1* and observing LB size in embryo hypocotyl cells. T2 seeds were preselected for reporter gene expression, imbibed in 0.1% agar at 4 °C over night and embryos were isolated by gentle squeezing between two glass slides. 100 μL of 0.1% agar containing the embryos was supplemented with 100 ng Nile Red (dissolved in acetone) and incubated in the dark at room temperature for 10 min before confocal microscopy as outlined above. Only embryos that appeared fully intact were observed more closely and the hypocotyl cells of three embryos per line were analyzed. The intensity of fluorescence emitted by the MCHERRY protein was negligible compared to the highly intense Nile Red stain, therefore permitting clear observation of stained LBs in the red part of the spectrum.

## Additional files

Additional file 1: LC-MS/MS dataset used for LBP identification. Specifications of LC-MS/MS measurements and data analysis are given and the data obtained are presented along with each filtering step in the identification of LBP candidates. (XLSX 7844 kb)

Additional file 2: Quantification of gene expression in *L. incisa*. Quantitative Real-time PCR results are shown for Figs. 2d, 4d, 5c and 7c. (XLSX 24 kb)

Additional file 3: Quantification of total fatty acids in *L. incisa*. GC measurements for *L. incisa* in conditions of nitrogen starvation and resupply for Figs. 2d, 4d, 5c and 7c are shown. (XLSX 40 kb)

Additional file 4: Confirmation of gene expression in transgenic *A. thaliana* lines. RNA was isolated from dry seeds and gene expression was analyzed by Reverse Transcriptase-PCR (RT-PCR) using primers detailed in Additional file 8. A, *A. thaliana oleo1* expressing LiMLDP. B, *A. thaliana sdp1/sdp1-L* expressing Li*LBP36*. C, *A. thaliana sdp1/sdp1-L* expressing Li*SDP1*. (PDF 1766 kb)

Additional file 5: Structural homology of LBP36 with yeast and fungal lipases. Phyre2 results are shown for the prediction of the LiLBP36 secondary structure and its alignment with known crystal structures. Additionally, an alignment of the predicted LiLBP36 structure (modeled on *Malassezia globosa* LIPASE1 using Phyre2) and Malassezia globosa LIPASE1 is shown. Only the structurally related section of LiLBP36 was aligned and the GXSXG motif as well as the catalytic aspartate are highlighted in green, with the two active residues being depicted as sticks. The 3D depiction was generated using PyMOL (The PyMOL Molecular Graphics System, Version 1.8 Schrödinger, LLC.). (PDF 1255 kb)

Additional file 6: Hypocotyl measurements used for calculating the effect of transgenes on postgerminative growth. Individual hypocotyl lengths are listed along with calculations for Figs. 6 and 8. EVC = Empty vector control. (XLSX 76 kb)

Additional file 7: Quantification of total fatty acids in complemented *A. thaliana* lines. GC measurements of seeds and seedlings for Fig. 8c are shown. (XLSX 127 kb)

Additional file 8: Primers used in this study. Primers used for RT-PCR and addition of restriction sites, qRT-PCR and confirmation of gene expression in transgenic lines are listed. Restriction sites are indicated capital letters. All primers were obtained from Sigma-Aldrich Chemie GmbH, Steinheim, Germany. CDS = Coding sequence. (DOCX 17 kb)

Additional file 9: pCambia 43.0 vector information. A map of vector features is shown in addition to the entire nucleotide sequence. (DOCX 88 kb)

## Abbreviations

*A. thaliana*: *Arabidopsis thaliana*
ARA: Arachidonic acid
DAG: Diacylglycerol
DTT: Dithiotreitol
DUF: Domain of unknown function
EDTA: Ethylenediaminetetraacetic acid
FPKM: Fragments per kilobase of transcript per million mapped reads
*L. incisa*: *Lobosphaera incisa*
LB: Lipid body
LBP: Lipid body protein
LC-MS/MS: Liquid chromatography-tandem mass spectrometry
LDAP: Lipid droplet associated protein
LDSP: Lipid droplet surface protein
MAG: Monoacylglycerol
MLDP: Major lipid droplet protein
MTBE: Methyl *tert*-butyl ether
*N. tabacum*: *Nicotiana tabacum*
napA: Napin A
NSAF: Normalized spectral abundance factor
OGP: Oil globule protein
SD: Standard deviation
SDP1: Sugar dependent 1
SDP1-L: SDP1-like
SDS: Sodium dodecyl sulfate
TAG: Triacylglycerol
TFA: Total fatty acids
