# Supplementary figures and images for: Analysis of the lipid body proteome of the oleaginous alga Lobosphaera incisa
Source: BMC Plant Biol. 2017 Jun 6;17:98. doi: 10.1186/s12870-017-1042-2 (PMC5461629; doi:10.1186/s12870-017-1042-2)

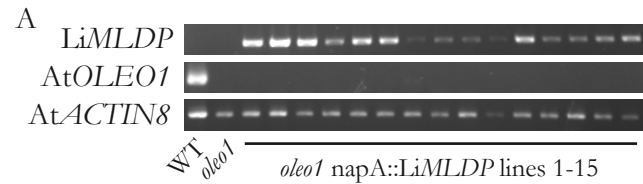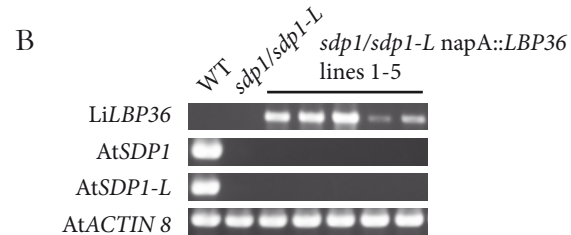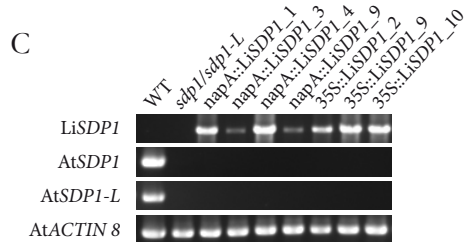

Supplement: Supplementary file 4 — Confirmation of gene expression in transgenic A. thaliana lines. RNA was isolated from dry seeds and gene expression was analyzed by Reverse Transcriptase-PCR (RT-PCR) using primers detailed in Additional file 8. A, A. thaliana oleo1 expressing LiMLDP. B, A. thaliana sdp1/sdp1-L expressing LiLBP36. C, A. thaliana sdp1/sdp1-L expressing LiSDP1. (PDF 1766 kb) [file 12870_2017_1042_MOESM4_ESM.pdf]
