# Supplementary material for: Analysis of the lipid body proteome of the oleaginous alga Lobosphaera incisa
Source: BMC Plant Biol. 2017 Jun 6;17:98. doi: 10.1186/s12870-017-1042-2 (PMC5461629; doi:10.1186/s12870-017-1042-2)

Phyre^2

Email	hsiegle@gwdg.de
Description	Undefined
Date	Wed Nov 30 10:59:22 GMT 2016
Unique Job ID	6f8220dc5c148320

Secondary structure and disorder prediction

1 . . . . 10 . . . . 20 . . . . 30 . . . . 40 . . . . 50 60

Sequence: M A S H D N L R A L R G P R E G R V D L R R P E N Y E S V Y D K M V D A F V W L A R T F C T W A V P R F Q P L L D F I V

Secondary structure

SS confidence

Disorder: ? ? ? ? ? ? ? ? ? ? ? ? ? ? ? ? ? ?

Disorder confidence

. . . . 70 . . . . 80 . . . . 90 . . . . 100 . . . . 110 120

Sequence: H H I C E P V F A R L T G S F Y P P K A V L H R G Q Q P V S F I S V T D P E F G V P L L D G K P L Y N P A N F P F D A T

Secondary structure

SS confidence

Disorder: ?

Disorder confidence

. . . . 130 . . . . 140 . . . . 150 . . . . 160 . . . . 170 180

Sequence: H A D A H G F H E W V A H F L I L C A K V A Y E R E A L I K M V V D R E F T G I K F V D S F P T E P K E A A N L T V E E

Secondary structure

SS confidence

Disorder: ? ? ? ? ? ? ? ?

Disorder confidence

. . . . 190 . . . . 200 . . . . 210 . . . . 220 . . . . 230 240

Sequence: K A H V M Q E A A F N R L M A G A D T A N P A M I K R R L G S E H Q Q L A N R V T A T L I P D T M A F M I T N D N A V I

Secondary structure

SS confidence

Disorder

Disorder confidence

. . . . 250 . . . . 260 . . . . 270 . . . . 280 . . . . 290 300

Sequence: L F F R G T E P H K L A Q W W S D C D L E L A V R T N A A G K V H Q G F W E A L F Y K A P P V N G K K E I P S V F N R I

Secondary structure

SS confidence

Disorder: ? ? ? ? ? ? ? ? ? ? ?

Disorder confidence

. . . . 310 . . . . 320 . . . . 330 . . . . 340 . . . . 350 360

Sequence: C A A L E R E T R G N H K R I Y V T G H S L G G G L T A M F A H T L A H P D T A K L P S T V G Y K D A H K L V D R V G G

Secondary structure

SS confidence

Disorder: ?

Disorder confidence

. . . . 370 . . . . 380 . . . . 390 . . . . 400 . . . . 410 420

Sequence: V Y T F A S P T A G N H A F C D F L V Y T Y G K N V K K G I G K D R L F R V A H S S D V V V K I P F G Q G Y R H H K L E

Secondary structure

SS confidence

Disorder: ? ? ? ? ? ? ?

Disorder confidence

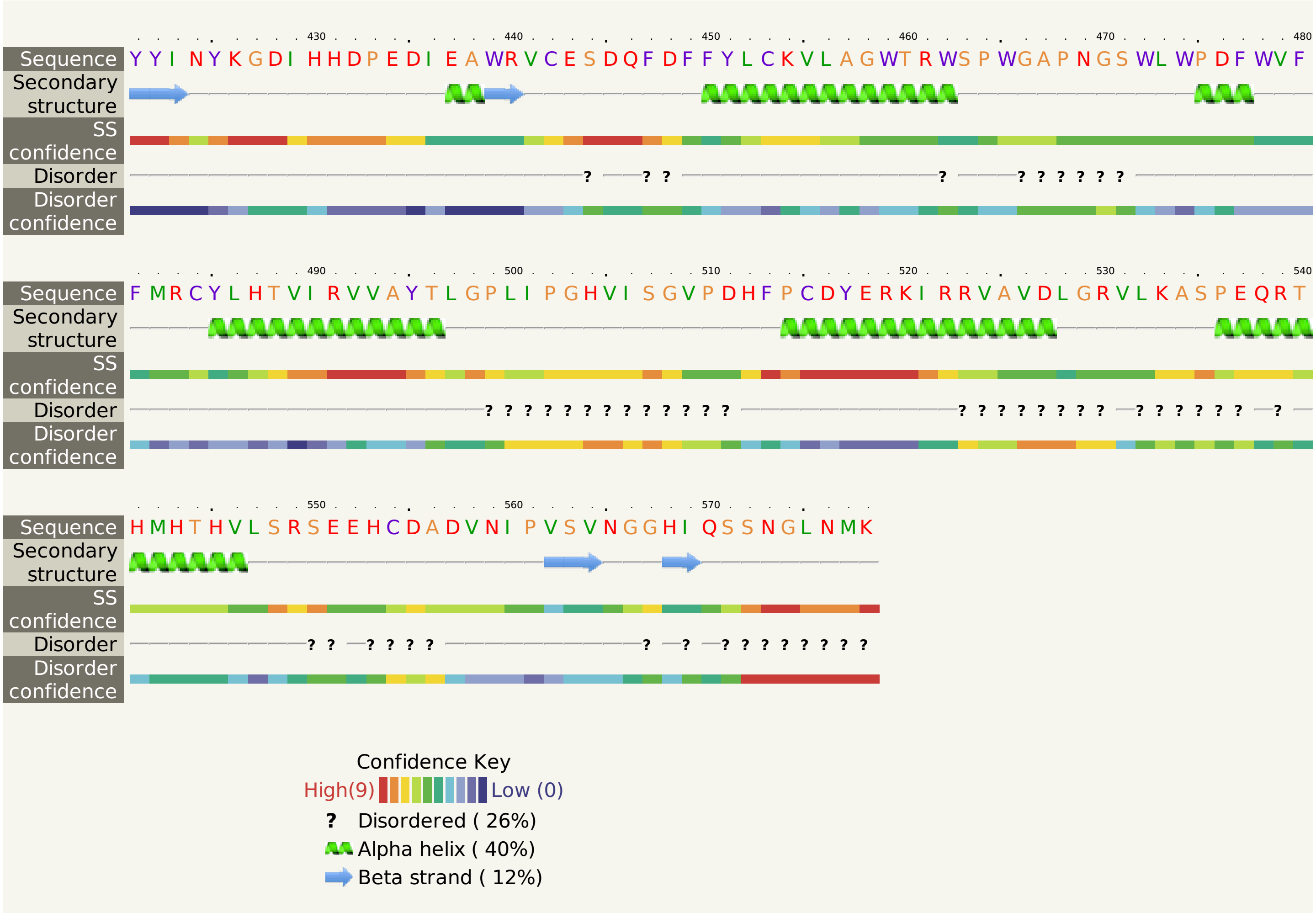

430 440 450 460 470 480
Sequence
YYINYKGDIHHDPEDIEAWRVCESDQFDFFYLCKVLAGWTRWSPWGAPNGSWLWPDFWVF
Secondary structure
SS confidence
Disorder
? ? ? ? ? ? ? ? ? ?
Disorder confidence
490 500 510 520 530 540
Sequence
FMRCYLHTVIRVVAYTLGPLIPGHVISGVPDHFPCDYERKIRRVAVDLGRVLKASPEQRT
Secondary structure
SS confidence
Disorder
? ?
Disorder confidence
550 560 570
Sequence
HMHTHVLSRSEEHCDADVNIPVSVNGGHIQSSNGLNMK
Secondary structure
SS confidence
Disorder
? ? ? ? ? ? ? ? ? ? ? ? ? ? ? ? ? ?
Disorder confidence
Confidence Key
High(9)
Low (0)
? Disordered (26%)
Alpha helix (40%)
Beta strand (12%)

Email	hsiegle@gwdg.de
Description	Undefined
Date	Wed Nov 30 10:59:22 GMT 2016
Unique Job ID	6f8220dc5c148320

Detailed template information

#	Template	Alignment Coverage	3D Model	Confidence	% i.d.	Template Information
1	c3o0dF_	Alignment	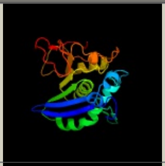	100.0	29	**PDB header:**hydrolase<br>**Chain:** F: **PDB Molecule:**triacylglycerol lipase;<br>**PDBTitle:** crystal structure of lip2 lipase from yarrowia lipolytica at 1.7 a2 resolution
2	c3uueA_	Alignment	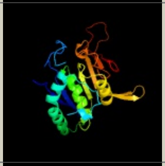	100.0	25	**PDB header:**hydrolase<br>**Chain:** A: **PDB Molecule:**lip1, secretory lipase (family 3);<br>**PDBTitle:** crystal structure of mono- and diacylglycerol lipase from malassezia2 globosa
3	c3ngmB_	Alignment	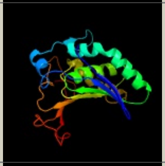	100.0	29	**PDB header:**hydrolase<br>**Chain:** B: **PDB Molecule:**extracellular lipase;<br>**PDBTitle:** crystal structure of lipase from gibberella zeae
4	d1uwca_	Alignment	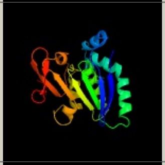	100.0	29	**Fold:**alpha/beta-Hydrolases<br>**Superfamily:**alpha/beta-Hydrolases<br>**Family:**Fungal lipases
5	d1tiaa_	Alignment	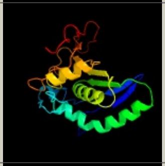	100.0	30	**Fold:**alpha/beta-Hydrolases<br>**Superfamily:**alpha/beta-Hydrolases<br>**Family:**Fungal lipases
6	c3g7nA_	Alignment	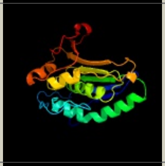	100.0	25	**PDB header:**hydrolase<br>**Chain:** A: **PDB Molecule:**lipase;<br>**PDBTitle:** crystal structure of a triacylglycerol lipase from penicillium2 expansum at 1.3
7	d1tiba_	Alignment	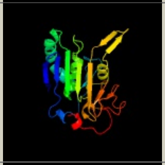	100.0	25	**Fold:**alpha/beta-Hydrolases<br>**Superfamily:**alpha/beta-Hydrolases<br>**Family:**Fungal lipases
8	d3tgla_	Alignment	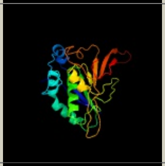	100.0	30	**Fold:**alpha/beta-Hydrolases<br>**Superfamily:**alpha/beta-Hydrolases<br>**Family:**Fungal lipases
9	d1lgya_	Alignment	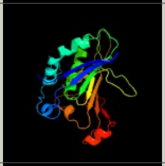	100.0	27	**Fold:**alpha/beta-Hydrolases<br>**Superfamily:**alpha/beta-Hydrolases<br>**Family:**Fungal lipases
10	c2yijA_	Alignment	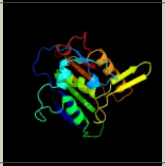	100.0	20	**PDB header:**hydrolase<br>**Chain:** A: **PDB Molecule:**phospholipase a1-iigamma;<br>**PDBTitle:** crystal structure of phospholipase a1
11	c4nfuB_	Alignment	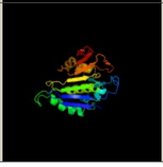	100.0	16	**PDB header:**signaling protein<br>**Chain:** B: **PDB Molecule:**senescence-associated carboxylesterase 101;<br>**PDBTitle:** structure of the central plant immunity signaling node eds1 in complex2 with its interaction partner sag101

12	c4nfuA_	Alignment	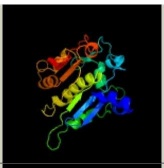	100.0	22	**PDB header:**signaling protein<br>**Chain:** A: **PDB Molecule:**eds1;<br>**PDBTitle:** structure of the central plant immunity signaling node eds1 in complex2 with its interaction partner sag101
13	c2oryA_	Alignment	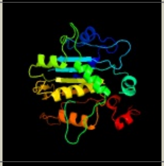	100.0	25	**PDB header:**hydrolase<br>**Chain:** A: **PDB Molecule:**lipase;<br>**PDBTitle:** crystal structure of m37 lipase
14	c4x96B_	Alignment	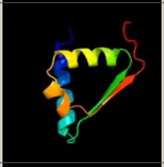	96.7	17	**PDB header:**transferase<br>**Chain:** B: **PDB Molecule:**phosphatidylcholine-sterol acyltransferase;<br>**PDBTitle:** low resolution crystal structure of lecithin:cholesterol2 acyltransferase (lcat; residues 21-397)
15	c4x91C_	Alignment	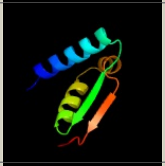	96.5	16	**PDB header:**transferase<br>**Chain:** C: **PDB Molecule:**group xv phospholipase a2;<br>**PDBTitle:** crystal structure of lysosomal phospholipase a2 in complex with2 isopropyl dodec-11-enylfluorophosphonate (idfp)
16	c4psdA_	Alignment	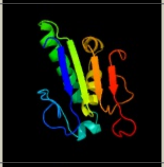	95.9	23	**PDB header:**hydrolase<br>**Chain:** A: **PDB Molecule:**carbohydrate esterase family 5;<br>**PDBTitle:** structure of trichoderma reesei cutinase native form.
17	c4z8zA_	Alignment	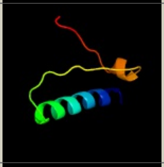	95.7	17	**PDB header:**unknown function<br>**Chain:** A: **PDB Molecule:**uncharacterized protein;<br>**PDBTitle:** crystal structure of the hypothetical protein from ruminiclostridium2 thermocellum atcc 27405
18	c3lp5A_	Alignment	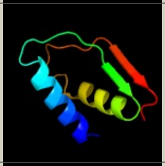	95.6	28	**PDB header:**hydrolase<br>**Chain:** A: **PDB Molecule:**putative cell surface hydrolase;<br>**PDBTitle:** the crystal structure of the putative cell surface hydrolase from2 lactobacillus plantarum wcfs1
19	c2zyiB_	Alignment	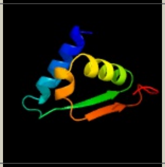	95.5	13	**PDB header:**hydrolase<br>**Chain:** B: **PDB Molecule:**lipase, putative;<br>**PDBTitle:** a. fulgidus lipase with fatty acid fragment and calcium
20	d1cexa_	Alignment	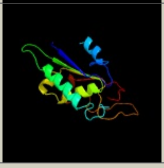	95.3	19	**Fold:**alpha/beta-Hydrolases<br>**Superfamily:**alpha/beta-Hydrolases<br>**Family:**Cutinase-like
21	c4r1dA_	Alignment	not modelled	94.9	22	**PDB header:**hydrolase/hydrolase inhibitor<br>**Chain:** A: **PDB Molecule:**uncharacterized protein;<br>**PDBTitle:** the crystal structure of tle4-tli4 complex
22	c5f2hA_	Alignment	not modelled	94.9	10	**PDB header:**unknown function<br>**Chain:** A: **PDB Molecule:**uncharacterized protein;<br>**PDBTitle:** 2.75 angstrom resolution crystal structure of uncharacterized protein2 from bacillus cereus atcc 10987
23	c3ds8A_	Alignment	not modelled	94.9	19	**PDB header:**structural genomics, unknown function<br>**Chain:** A: **PDB Molecule:**lin2722 protein;<br>**PDBTitle:** the crysatl structure of the gene lin2722 products from listeria2 innocua
24	d1uxoa_	Alignment	not modelled	94.8	22	**Fold:**alpha/beta-Hydrolases<br>**Superfamily:**alpha/beta-Hydrolases<br>**Family:**YdeN-like
25	d1cvla_	Alignment	not modelled	94.5	31	**Fold:**alpha/beta-Hydrolases<br>**Superfamily:**alpha/beta-Hydrolases<br>**Family:**Bacterial lipase
26	d1ex9a_	Alignment	not modelled	94.4	24	**Fold:**alpha/beta-Hydrolases<br>**Superfamily:**alpha/beta-Hydrolases<br>**Family:**Bacterial lipase
27	c4qnnC_	Alignment	not modelled	94.3	17	**PDB header:**hydrolase<br>**Chain:** C: **PDB Molecule:**phospholipase a 1 from hornet(vespa basalis) venom;<br>**PDBTitle:** crystal structure of phospholipase a 1 from hornet(vespa basalis)2 venom
28	d1ispa_	Alignment	not modelled	94.3	19	**Fold:**alpha/beta-Hydrolases<br>**Superfamily:**alpha/beta-Hydrolases<br>**Family:**Bacterial lipase
						**PDB header:**hydrolase

29	c3d0kA_	Alignment	not modelled	94.2	21	**Chain:** A: **PDB Molecule:**putative poly(3-hydroxybutyrate) depolymerase lpqc; **PDBTitle:** crystal structure of the lpqc, poly(3-hydroxybutyrate) depolymerase2 from bordetella parapertussis
30	d1tcaa_	Alignment	not modelled	94.2	21	**Fold:**alpha/beta-Hydrolases **Superfamily:**alpha/beta-Hydrolases **Family:**Fungal lipases
31	c2h7xA_	Alignment	not modelled	94.0	23	**PDB header:**hydrolase **Chain:** A: **PDB Molecule:**type i polyketide synthase pikaiv; **PDBTitle:** pikromycin thioesterase adduct with reduced triketide2 affinity label
32	d1sfra_	Alignment	not modelled	94.0	13	**Fold:**alpha/beta-Hydrolases **Superfamily:**alpha/beta-Hydrolases **Family:**Mycobacterial antigens
33	c4gw3A_	Alignment	not modelled	93.9	25	**PDB header:**hydrolase **Chain:** A: **PDB Molecule:**putative lipase; **PDBTitle:** crystal structure of the lipase from proteus mirabilis
34	d2h1ia1	Alignment	not modelled	93.8	17	**Fold:**alpha/beta-Hydrolases **Superfamily:**alpha/beta-Hydrolases **Family:**Carboxylesterase/thioesterase 1
35	c3fleB_	Alignment	not modelled	93.8	17	**PDB header:**structural genomics, unknown function **Chain:** B: **PDB Molecule:**se_1780 protein; **PDBTitle:** se_1780 protein of unknown function from staphylococcus epidermidis.
36	d2jbwa1	Alignment	not modelled	93.8	17	**Fold:**alpha/beta-Hydrolases **Superfamily:**alpha/beta-Hydrolases **Family:**2,6-dihydropseudooxynicotine hydrolase-like
37	d2h7xa1	Alignment	not modelled	93.8	23	**Fold:**alpha/beta-Hydrolases **Superfamily:**alpha/beta-Hydrolases **Family:**Thioesterase domain of polypeptide, polyketide and fatty acid synthases
38	c2h1iA_	Alignment	not modelled	93.7	18	**PDB header:**hydrolase **Chain:** A: **PDB Molecule:**carboxylesterase; **PDBTitle:** crystal structure of the bacillus cereus carboxylesterase
39	c1pjaA_	Alignment	not modelled	93.7	11	**PDB header:**hydrolase **Chain:** A: **PDB Molecule:**palmitoyl-protein thioesterase 2 precursor; **PDBTitle:** the crystal structure of palmitoyl protein thioesterase-2 reveals the2 basis for divergent substrate specificities of the two lysosomal3 thioesterases (ppt1 and ppt2)
40	d1pjaa_	Alignment	not modelled	93.7	11	**Fold:**alpha/beta-Hydrolases **Superfamily:**alpha/beta-Hydrolases **Family:**Thioesterases
41	c2y6vB_	Alignment	not modelled	93.6	10	**PDB header:**hydrolase **Chain:** B: **PDB Molecule:**peroxisomal membrane protein lpx1; **PDBTitle:** peroxisomal alpha-beta-hydrolase lpx1 (yor084w) from2 saccharomyces cerevisiae (crystal form i)
42	d1ei9a_	Alignment	not modelled	93.6	19	**Fold:**alpha/beta-Hydrolases **Superfamily:**alpha/beta-Hydrolases **Family:**Thioesterases
43	c2rauA_	Alignment	not modelled	93.5	18	**PDB header:**hydrolase **Chain:** A: **PDB Molecule:**putative esterase; **PDBTitle:** crystal structure of a putative lipase (np_343859.1) from sulfolobus2 solfataricus at 1.85 a resolution
44	c3lcrA_	Alignment	not modelled	93.5	20	**PDB header:**hydrolase **Chain:** A: **PDB Molecule:**tautomycetin biosynthetic pks; **PDBTitle:** thioesterase from tautomycetin biosynthhetic pathway
45	c1qgeD_	Alignment	not modelled	93.4	22	**PDB header:**hydrolase **Chain:** D: **PDB Molecule:**protein (triacylglycerol hydrolase); **PDBTitle:** new crystal form of pseudomonas glumae (formerly chromobacterium2 viscosum atcc 6918) lipase
46	d1r88a_	Alignment	not modelled	93.0	8	**Fold:**alpha/beta-Hydrolases **Superfamily:**alpha/beta-Hydrolases **Family:**Mycobacterial antigens
47	c3mveB_	Alignment	not modelled	93.0	11	**PDB header:**lyase **Chain:** B: **PDB Molecule:**upf0255 protein vv1_0328; **PDBTitle:** crystal structure of a novel pyruvate decarboxylase
48	c1mo2A_	Alignment	not modelled	93.0	25	**PDB header:**transferase **Chain:** A: **PDB Molecule:**erythronolide synthase, modules 5 and 6; **PDBTitle:** thioesterase domain from 6-deoxyerythronolide synthase2 (debs te), ph 8.5
49	d1mo2a_	Alignment	not modelled	93.0	25	**Fold:**alpha/beta-Hydrolases **Superfamily:**alpha/beta-Hydrolases **Family:**Thioesterase domain of polypeptide, polyketide and fatty acid synthases
50	c2jbwB_	Alignment	not modelled	92.9	23	**PDB header:**hydrolase **Chain:** B: **PDB Molecule:**2,6-dihydroxy-pseudo-oxynicotine hydrolase; **PDBTitle:** crystal structure of the 2,6-dihydroxy-pseudo-oxynicotine2 hydrolase.
51	c4fleA_	Alignment	not modelled	92.7	15	**PDB header:**hydrolase **Chain:** A: **PDB Molecule:**esterase; **PDBTitle:** crystal structure of the esterase yqia (ye3661) from yersinia2 enterocolitica, northeast structural genomics consortium target yer85
52	c3bdvB_	Alignment	not modelled	92.7	22	**PDB header:**hydrolase **Chain:** B: **PDB Molecule:**uncharacterized protein duf1234; **PDBTitle:** crystal structure of a putative yden-like hydrolase (eca3091) from2 pectobacterium atrosepticum scri1043 at 1.66 a resolution
53	d3b5ea1	Alignment	not modelled	92.6	16	**Fold:**alpha/beta-Hydrolases **Superfamily:**alpha/beta-Hydrolases **Family:**Carboxylesterase/thioesterase 1
54	d1k8qa_	Alignment	not modelled	92.5	22	**Fold:**alpha/beta-Hydrolases **Superfamily:**alpha/beta-Hydrolases

						**Family:**Gastric lipase
55	d1xkta_	Alignment	not modelled	92.5	13	**Fold:**alpha/beta-Hydrolases<br>**Superfamily:**alpha/beta-Hydrolases<br>**Family:**Thioesterase domain of polypeptide, polyketide and fatty acid synthases
56	d4lipd_	Alignment	not modelled	92.4	30	**Fold:**alpha/beta-Hydrolases<br>**Superfamily:**alpha/beta-Hydrolases<br>**Family:**Bacterial lipase
57	c3picB_	Alignment	not modelled	92.4	31	**PDB header:**hydrolase<br>**Chain:** B: **PDB Molecule:**cip2;<br>**PDBTitle:** glucuronoyl esterase catalytic domain (cip2_ge) from hypocrea jecorina
58	c2qruA_	Alignment	not modelled	92.4	18	**PDB header:**structural genomics, unknown function<br>**Chain:** A: **PDB Molecule:**uncharacterized protein;<br>**PDBTitle:** crystal structure of an alpha/beta hydrolase superfamily protein from2 enterococcus faecalis
59	d1lpbb2	Alignment	not modelled	92.3	18	**Fold:**alpha/beta-Hydrolases<br>**Superfamily:**alpha/beta-Hydrolases<br>**Family:**Pancreatic lipase, N-terminal domain
60	c3l80A_	Alignment	not modelled	92.2	13	**PDB header:**hydrolase<br>**Chain:** A: **PDB Molecule:**putative uncharacterized protein smu.1393c;<br>**PDBTitle:** crystal structure of smu.1393c from streptococcus mutans ua159
61	d1hlga_	Alignment	not modelled	92.0	24	**Fold:**alpha/beta-Hydrolases<br>**Superfamily:**alpha/beta-Hydrolases<br>**Family:**Gastric lipase
62	d1bu8a2	Alignment	not modelled	91.8	21	**Fold:**alpha/beta-Hydrolases<br>**Superfamily:**alpha/beta-Hydrolases<br>**Family:**Pancreatic lipase, N-terminal domain
63	c2vavL_	Alignment	not modelled	91.7	20	**PDB header:**transferase<br>**Chain:** L: **PDB Molecule:**acetyl-coa--deacetylcephalosporin c<br>**PDBTitle:** crystal structure of deacetylcephalosporin c2 acetyltransferase (dac-soak)
64	c2veoA_	Alignment	not modelled	91.6	19	**PDB header:**hydrolase<br>**Chain:** A: **PDB Molecule:**lipase a;<br>**PDBTitle:** x-ray structure of candida antarctica lipase a in its closed state.
65	c4h0cA_	Alignment	not modelled	91.6	11	**PDB header:**hydrolase<br>**Chain:** A: **PDB Molecule:**phospholipase/carboxylesterase;<br>**PDBTitle:** crystal structure of phospholipase/carboxylesterase from dyadobacter2 fermentans dsm 18053
66	c3h2iA_	Alignment	not modelled	91.5	15	**PDB header:**hydrolase<br>**Chain:** A: **PDB Molecule:**esterase;<br>**PDBTitle:** crystal structure of n228w mutant of the rice cell wall2 degrading esterase lipa from xanthomonas oryzae
67	c3wlaA_	Alignment	not modelled	91.5	13	**PDB header:**hydrolase<br>**Chain:** A: **PDB Molecule:**oxidized polyvinyl alcohol hydrolase;<br>**PDBTitle:** crystal structure of soph native
68	d1f0na_	Alignment	not modelled	91.4	13	**Fold:**alpha/beta-Hydrolases<br>**Superfamily:**alpha/beta-Hydrolases<br>**Family:**Mycobacterial antigens
69	c3i28A_	Alignment	not modelled	91.4	25	**PDB header:**hydrolase<br>**Chain:** A: **PDB Molecule:**epoxide hydrolase 2;<br>**PDBTitle:** crystal structure of soluble epoxide hydrolase
70	c2cbgA_	Alignment	not modelled	91.4	14	**PDB header:**hydrolase<br>**Chain:** A: **PDB Molecule:**fengycin synthetase;<br>**PDBTitle:** crystal structure of the pmsf-inhibited thioesterase domain2 of the fengycin biosynthesis cluster
71	d1g66a_	Alignment	not modelled	91.2	15	**Fold:**alpha/beta-Hydrolases<br>**Superfamily:**alpha/beta-Hydrolases<br>**Family:**Cutinase-like
72	c4eziA_	Alignment	not modelled	91.2	24	**PDB header:**unknown function<br>**Chain:** A: **PDB Molecule:**uncharacterized protein;<br>**PDBTitle:** crystal structure of a putative hydrolase (lpg1103) from legionella2 pneumophila subsp. pneumophila str. philadelphia 1 at 1.15 a3 resolution
73	c3k2iA_	Alignment	not modelled	91.1	19	**PDB header:**hydrolase<br>**Chain:** A: **PDB Molecule:**acyl-coenzyme a thioesterase 4;<br>**PDBTitle:** human acyl-coenzyme a thioesterase 4
74	d1gpla2	Alignment	not modelled	91.0	22	**Fold:**alpha/beta-Hydrolases<br>**Superfamily:**alpha/beta-Hydrolases<br>**Family:**Pancreatic lipase, N-terminal domain
75	c3ajaA_	Alignment	not modelled	90.9	14	**PDB header:**hydrolase<br>**Chain:** A: **PDB Molecule:**putative uncharacterized protein;<br>**PDBTitle:** crystal structure of msmeg_6394
76	c4ao6A_	Alignment	not modelled	90.8	13	**PDB header:**hydrolase<br>**Chain:** A: **PDB Molecule:**esterase;<br>**PDBTitle:** native structure of a novel cold-adapted esterase from an2 arctic intertidal metagenomic library
77	c3fcxA_	Alignment	not modelled	90.8	18	**PDB header:**hydrolase<br>**Chain:** A: **PDB Molecule:**s-formylglutathione hydrolase;<br>**PDBTitle:** crystal structure of human esterase d
78	c3wydA_	Alignment	not modelled	90.8	35	**PDB header:**hydrolase<br>**Chain:** A: **PDB Molecule:**lc-est1c;<br>**PDBTitle:** c-terminal esterase domain of lc-est1
79	c3og9A_	Alignment	not modelled	90.8	12	**PDB header:**hydrolase<br>**Chain:** A: **PDB Molecule:**protein yahd a copper inducible hydrolase;<br>**PDBTitle:** structure of yahd with malic acid
80	c3g8yA_	Alignment	not modelled	90.8	17	**PDB header:**hydrolase<br>**Chain:** A: **PDB Molecule:**susd/ragb-associated esterase-like protein;<br>**PDBTitle:** crystal structure of a putative hydrolase (bvu_4111) from bacteroides2 vulgatus atcc 8482 at 1.90 a resolution

81	c4g4gA_	Alignment	not modelled	90.7	27	**PDB header:**hydrolase<br>**Chain:** A: **PDB Molecule:**4-o-methyl-glucuronoyl methylesterase;<br>**PDBTitle:** crystal structure of recombinant glucuronoyl esterase from2 sporotrichum thermophile determined at 1.55 a resolution
82	c3h04A_	Alignment	not modelled	90.6	16	**PDB header:**structural genomics, unknown function<br>**Chain:** A: **PDB Molecule:**uncharacterized protein;<br>**PDBTitle:** the crystal structure of the protein with unknown function from2 staphylococcus aureus subsp. aureus mu50
83	c3fnbB_	Alignment	not modelled	90.5	22	**PDB header:**hydrolase<br>**Chain:** B: **PDB Molecule:**acylaminoacyl peptidase smu_737;<br>**PDBTitle:** crystal structure of acylaminoacyl peptidase smu_737 from2 streptococcus mutans ua159
84	d1dqza_	Alignment	not modelled	90.4	17	**Fold:**alpha/beta-Hydrolases<br>**Superfamily:**alpha/beta-Hydrolases<br>**Family:**Mycobacterial antigens
85	c3qmwD_	Alignment	not modelled	90.3	28	**PDB header:**hydrolase<br>**Chain:** D: **PDB Molecule:**thioesterase;<br>**PDBTitle:** redj with peg molecule bound in the active site
86	c3gbsA_	Alignment	not modelled	90.3	16	**PDB header:**hydrolase<br>**Chain:** A: **PDB Molecule:**cutinase 1;<br>**PDBTitle:** crystal structure of aspergillus oryzae cutinase
87	c2qs9A_	Alignment	not modelled	90.1	19	**PDB header:**structural protein<br>**Chain:** A: **PDB Molecule:**retinoblastoma-binding protein 9;<br>**PDBTitle:** crystal structure of the human retinoblastoma-binding2 protein 9 (rbbp-9). nesg target hr2978
88	d1qoza_	Alignment	not modelled	90.1	17	**Fold:**alpha/beta-Hydrolases<br>**Superfamily:**alpha/beta-Hydrolases<br>**Family:**Cutinase-like
89	c2q0xA_	Alignment	not modelled	90.0	11	**PDB header:**structural genomics, unknown function<br>**Chain:** A: **PDB Molecule:**uncharacterized protein;<br>**PDBTitle:** alpha/beta hydrolase fold protein of unknown function
90	c3flaB_	Alignment	not modelled	90.0	17	**PDB header:**hydrolase<br>**Chain:** B: **PDB Molecule:**rifr;<br>**PDBTitle:** rifr - type ii thioesterase from rifamycin nrps/pks biosynthetic2 pathway - form 1
91	c2r8bA_	Alignment	not modelled	90.0	11	**PDB header:**structural genomics, unknown function<br>**Chain:** A: **PDB Molecule:**uncharacterized protein atu2452;<br>**PDBTitle:** the crystal structure of the protein atu2452 of unknown function from2 agrobacterium tumefaciens str. c58
92	c2czqB_	Alignment	not modelled	89.9	16	**PDB header:**hydrolase<br>**Chain:** B: **PDB Molecule:**cutinase-like protein;<br>**PDBTitle:** a novel cutinase-like protein from cryptococcus sp.
93	c2qjwA_	Alignment	not modelled	89.7	18	**PDB header:**hydrolase<br>**Chain:** A: **PDB Molecule:**uncharacterized protein xcc1541;<br>**PDBTitle:** crystal structure of a putative hydrolase of the alpha/beta2 superfamily (xcc1541) from xanthomonas campestris pv. campestris at3 1.35 a resolution
94	c3dd5F_	Alignment	not modelled	89.6	19	**PDB header:**hydrolase<br>**Chain:** F: **PDB Molecule:**cutinase;<br>**PDBTitle:** glomerella cingulata e600-cutinase complex
95	c4xjvA_	Alignment	not modelled	89.2	19	**PDB header:**hydrolase<br>**Chain:** A: **PDB Molecule:**s-acyl fatty acid synthase thioesterase, medium chain;<br>**PDBTitle:** crystal structure of human thioesterase 2
96	d1etha2	Alignment	not modelled	89.2	18	**Fold:**alpha/beta-Hydrolases<br>**Superfamily:**alpha/beta-Hydrolases<br>**Family:**Pancreatic lipase, N-terminal domain
97	c3hlkB_	Alignment	not modelled	89.1	24	**PDB header:**hydrolase<br>**Chain:** B: **PDB Molecule:**acyl-coenzyme a thioesterase 2, mitochondrial;<br>**PDBTitle:** crystal structure of human mitochondrial acyl-coa2 thioesterase (acot2)
98	c3d59B_	Alignment	not modelled	88.8	35	**PDB header:**hydrolase<br>**Chain:** B: **PDB Molecule:**platelet-activating factor acetylhydrolase;<br>**PDBTitle:** crystal structure of human plasma platelet activating2 factor acetylhydrolase
99	d1rp1a2	Alignment	not modelled	88.8	16	**Fold:**alpha/beta-Hydrolases<br>**Superfamily:**alpha/beta-Hydrolases<br>**Family:**Pancreatic lipase, N-terminal domain
100	c1cr6A_	Alignment	not modelled	88.6	10	**PDB header:**hydrolase<br>**Chain:** A: **PDB Molecule:**epoxide hydrolase;<br>**PDBTitle:** crystal structure of murine soluble epoxide hydrolase2 complexed with cpu inhibitor
101	d2r8ba1	Alignment	not modelled	88.6	11	**Fold:**alpha/beta-Hydrolases<br>**Superfamily:**alpha/beta-Hydrolases<br>**Family:**Carboxylesterase/thioesterase 1
102	c4eb0A_	Alignment	not modelled	88.5	50	**PDB header:**hydrolase<br>**Chain:** A: **PDB Molecule:**lcc;<br>**PDBTitle:** crystal structure of leaf-branch compost bacterial cutinase homolog
103	c4wfiA_	Alignment	not modelled	88.5	40	**PDB header:**hydrolase<br>**Chain:** A: **PDB Molecule:**cutinase;<br>**PDBTitle:** crystal structure of pet-degrading cutinase cut190 s226p mutant in2 ca(2+)-free state
104	c5d6oB_	Alignment	not modelled	88.3	21	**PDB header:**transferase<br>**Chain:** B: **PDB Molecule:**homoserine o-acetyltransferase;<br>**PDBTitle:** orthorhombic crystal structure of an acetylester hydrolase from2 corynebacterium glutamicum
105	d1jmkc_	Alignment	not modelled	88.2	16	**Fold:**alpha/beta-Hydrolases<br>**Superfamily:**alpha/beta-Hydrolases<br>**Family:**Thioesterase domain of polypeptide, polyketide and fatty acid synthases
106	c5cxxC_	Alignment	not modelled	88.0	13	**PDB header:**hydrolase<br>**Chain:** C: **PDB Molecule:**ferulic acid esterase, amce1/fae1a;<br>**PDBTitle:** structure of a ce1 ferulic acid esterase, amce1/fae1a, from2 anaeromyces mucronatus in complex with ferulic acid

107	c3visB_	Alignment	not modelled	87.9	50	**PDB header:**hydrolase<br>**Chain:** B: **PDB Molecule:**esterase;<br>**PDBTitle:** crystal structure of cutinase est119 from thermobifida alba ahk119
108	c4oylC_	Alignment	not modelled	87.9	18	**PDB header:**hydrolase<br>**Chain:** C: **PDB Molecule:**cutinase;<br>**PDBTitle:** humicola insolens cutinase in complex with mono-ethylphosphate
109	d1jfra_	Alignment	not modelled	87.6	45	**Fold:**alpha/beta-Hydrolases<br>**Superfamily:**alpha/beta-Hydrolases<br>**Family:**Lipase
110	d1hpla2	Alignment	not modelled	87.2	16	**Fold:**alpha/beta-Hydrolases<br>**Superfamily:**alpha/beta-Hydrolases<br>**Family:**Pancreatic lipase, N-terminal domain
111	c2uz0B_	Alignment	not modelled	87.1	13	**PDB header:**hydrolase<br>**Chain:** B: **PDB Molecule:**tributyrin esterase;<br>**PDBTitle:** the crystal crystal structure of the esta protein, a2 virulence factor esta protein from streptococcus pneumonia
112	d1pv1a_	Alignment	not modelled	87.0	18	**Fold:**alpha/beta-Hydrolases<br>**Superfamily:**alpha/beta-Hydrolases<br>**Family:**Hypothetical esterase YJL068C
113	c4qloA_	Alignment	not modelled	87.0	24	**PDB header:**transferase<br>**Chain:** A: **PDB Molecule:**homoserine o-acetyltransferase;<br>**PDBTitle:** crystal structure of homoserine o-acetyltransferase from2 staphylococcus aureus
114	c3i6yA_	Alignment	not modelled	86.9	21	**PDB header:**hydrolase<br>**Chain:** A: **PDB Molecule:**esterase apc40077;<br>**PDBTitle:** structure of an esterase from the oil-degrading bacterium oleispira2 antarctica
115	c2hu7A_	Alignment	not modelled	86.9	18	**PDB header:**hydrolase<br>**Chain:** A: **PDB Molecule:**acylamino-acid-releasing enzyme;<br>**PDBTitle:** binding of inhibitors by acylaminoacyl peptidase
116	c3jyhD_	Alignment	not modelled	86.8	29	**PDB header:**hydrolase<br>**Chain:** D: **PDB Molecule:**dipeptidyl-peptidase 2;<br>**PDBTitle:** human dipeptidyl peptidase dpp7
117	c3doiA_	Alignment	not modelled	86.8	25	**PDB header:**hydrolase<br>**Chain:** A: **PDB Molecule:**esterase;<br>**PDBTitle:** crystal structure of a thermostable esterase complex with2 paraoxon
118	c3e4dD_	Alignment	not modelled	86.7	22	**PDB header:**hydrolase<br>**Chain:** D: **PDB Molecule:**esterase d;<br>**PDBTitle:** structural and kinetic study of an s-formylglutathione2 hydrolase from agrobacterium tumefaciens
119	c4fhzA_	Alignment	not modelled	86.5	12	**PDB header:**hydrolase<br>**Chain:** A: **PDB Molecule:**phospholipase/carboxylesterase;<br>**PDBTitle:** crystal structure of a carboxyl esterase at 2.0 angstrom resolution
120	c4b6gA_	Alignment	not modelled	86.5	19	**PDB header:**hydrolase<br>**Chain:** A: **PDB Molecule:**putative esterase;<br>**PDBTitle:** the crystal structure of the neisserial esterase d.

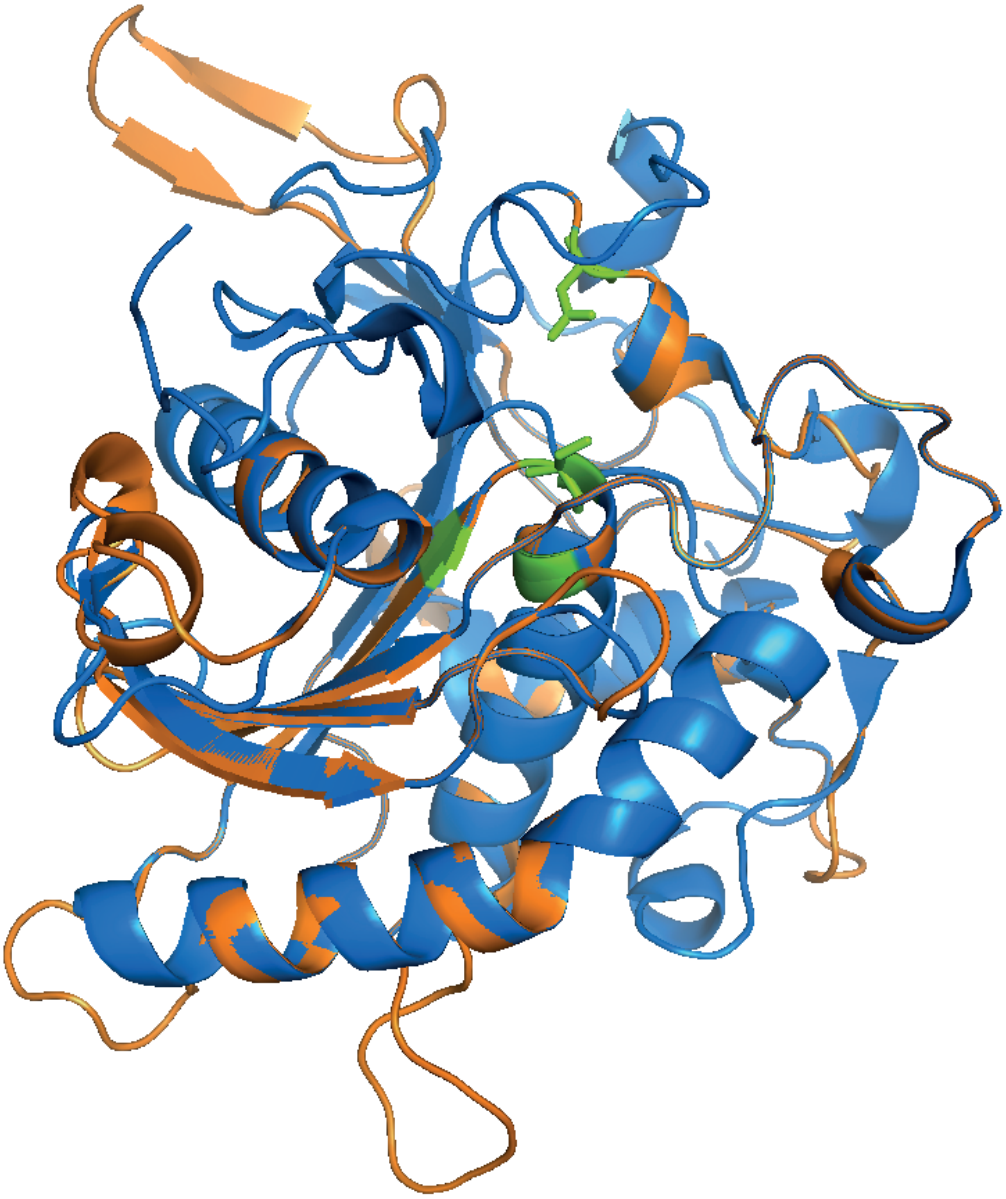

Supplement: Supplementary file 5 — Structural homology of LBP36 with yeast and fungal lipases. Phyre2 results are shown for the prediction of the LiLBP36 secondary structure and its alignment with known crystal structures. Additionally, an alignment of the predicted LiLBP36 structure (modeled on Malassezia globosa LIPASE1 using Phyre2) and Malassezia globosa LIPASE1 is shown. Only the structurally related section of LiLBP36 was aligned and the GXSXG motif as well as the catalytic aspartate are highlighted in green, with the two active residues being depicted as sticks. The 3D depiction was generated using PyMOL (The PyMOL Molecular Graphics System, Version 1.8 Schrödinger, LLC.). (PDF 1255 kb) [file 12870_2017_1042_MOESM5_ESM.pdf]
