# Supplementary material for: Analysis of the lipid body proteome of the oleaginous alga Lobosphaera incisa
Source: BMC Plant Biol. 2017 Jun 6;17:98. doi: 10.1186/s12870-017-1042-2 (PMC5461629; doi:10.1186/s12870-017-1042-2)
Supplement: Supplementary file 8 — Primers used in this study. Primers used for RT-PCR and addition of restriction sites, qRT-PCR and confirmation of gene expression in transgenic lines are listed. Restriction sites are indicated capital letters. All primers were obtained from Sigma-Aldrich Chemie GmbH, Steinheim, Germany. CDS = Coding sequence. (DOCX 17 kb) [file 12870_2017_1042_MOESM8_ESM.docx]

Amplification of coding sequences from cDNA and addition of restriction sites

| Target sequence | Primer nucleotide sequence | Restriction site | Target vector |
| --- | --- | --- | --- |
| g555 (Li*OGP*) CDS | ataGGATCCatgggtgctgagccagtg | BamHI | pUC-LAT52-mVenusC |
|  | ataGTCGACgtgagcgccgttcacctc | SalI |  |
|  | ataGTCGACatgggtgctgagccagtg | SalI | pENTRY‑E |
|  | ataGGATCCtcagtgagcgccgttcac | BamHI |  |
| g9864 CDS | ataGGATCCatggcatcagcagttgcaac | BamHI | pUC-LAT52-mVenusC |
|  | ataCCCGGGcacggcttttttggctggc | SmaI |  |
| g9192 (Li*SDP1*) CDS | ggaGGATCCatgaaacctctgacttaccaaac | BamHI | pUC-LAT52-mVenusC |
|  | ataGTCGACtggcgcgatgacgtccag | SalI |  |
|  | ataGTCGACatgaaacctctgacttaccaaac | SalI | pENTRY‑E |
|  | ataGGATCCtcatggcgcgatgacgtc | BamHI |  |
| g9582 CDS | ataGGATCCatgcaagacccgtaccaacg | BamHI | pUC-LAT52-mVenusC |
|  | ataCTCGAGgggcaaaaacagctggtagt | XhoI |  |
| g13714 CDS | ataGGATCCatgcgtgccgcagcaagc | BamHI | pUC-LAT52-mVenusC |
|  | ataCTCGAGtgccgccgctgtcaacgc | XhoI |  |
| g4703 CDS | ataGGATCCatggctacccggcaaccc | BamHI | pUC-LAT52-mVenusC |
|  | ataCCCGGGggtgtgagcagcggcgac | SmaI |  |
| g13209 CDS | ataCTCGAGatggtctctctgtggtacttgg | XhoI | pUC-LAT52-mVenusC |
|  | ataCCCGGGcttaaactcacaagtgagctgc | SmaI |  |
| g12144 CDS | ataGGATCCatggagccggagcttggt | BamHI | pUC-LAT52-mVenusC |
|  | ataGTCGACtgccacgcgcttggtggag | SalI |  |
| g13747 CDS | agaGGATCCatgactaagtccacctcatccc | BamHI | pUC-LAT52-mVenusC |
|  | ataGTCGACctgttcagcgctagcggc | SalI |  |
| g13945 (Li*LBP*36) CDS | ataGGATCCatggcatctcatgacaacctg | BamHI | pUC-LAT52-mVenusC |
|  | ataCTCGAGtttcatgtttaggccattgctg | XhoI |  |
|  | ataACATGTatggcatctcatgacaacctg | PciI | pENTRY‑E |
|  | ataGGATCCtcatttcatgtttaggccattgc | BamHI |  |
| g14373 CDS | ggcCTCGAGatgtctgtccgtcagccg | XhoI | pUC-LAT52-mVenusC |
|  | ataCCCGGGtgccgctgcagtcgccggc | SmaI |  |
| g15430 (LiL*BP62*) CDS | ataGGATCCatgtataacgcagacgggtccat | BamHI | pUC-LAT52-mVenusC |
|  | ataGTCGACccatccaaagctgaacgtgc | SalI |  |
|  | ataGTCGACatgtataacgcagacgggtc | SalI | pENTRY‑E |
|  | ataGGATCCtcaccatccaaagctgaacg | BamHI |  |

Confirmation of gene expression

| Target sequence | Primer nucleotide sequence |
| --- | --- |
| *A. thaliana ACTIN8* CDS | atggccgatgctgatgacattcaacct |
|  | ttagaagcattttctgtggacaatgcctg |
| *A. thaliana* *SDP1* CDS | accgtttgctccccaagaaa |
|  | cttgagcctccgcatatggt |
| *A. thaliana* *SDP1‑L* CDS | tcattcctcggtgggttagc |
|  | tacagaccccacgcttgaac |
| *A. thaliana OLEO1* CDS | atggcggatacagctagagg |
|  | ttaagtagtgtgctggccac |
| g13945 (Li*LBP*36) CDS | atggcatctcatgacaacctg |
|  | ttatttcatgtttaggccattg |
| g9192 (Li*SDP1*) CDS | atgaaacctctgacttaccaaacgggtcgc |
|  | tcatggcgcgatgacgtccagcgcctggcc |
| g555 (Li*OGP*) CDS | atgggtgctgagccagtg |
|  | ttagtgagcgccgttcac |

qRT-PCR

| Target sequence | Primer nucleotide sequence |
| --- | --- |
| g555 (Li*OGP*) CDS | tgtctacagtactggcaagagc |
|  | tgttccatactcgctgactgtc |
| g5830 (*RIBOSOMAL PROTEIN S21*) CDS | caacgtagacaaggccttttcc |
|  | tctccttctggttgagcactc |
| g9192 (Li*SDP1*) CDS | ggtgttgtcaagacgctctttg |
|  | ttggttgcgatgatggatgc |
| g13945 (Li*LBP*36) CDS | atgctcacaaactcgtggac |
|  | ttgccaatgcccttcttgac |
| g15430 (LiL*BP62*) CDS | acatctttggcaaccacagc |
|  | atcgctgatgtcacggactc |
