# Supplementary material for: Analysis of the lipid body proteome of the oleaginous alga Lobosphaera incisa
Source: BMC Plant Biol. 2017 Jun 6;17:98. doi: 10.1186/s12870-017-1042-2 (PMC5461629; doi:10.1186/s12870-017-1042-2)
Supplement: Supplementary file 9 — pCambia 43.0 vector information. A map of vector features is shown in addition to the entire nucleotide sequence. (DOCX 88 kb) [file 12870_2017_1042_MOESM9_ESM.docx]

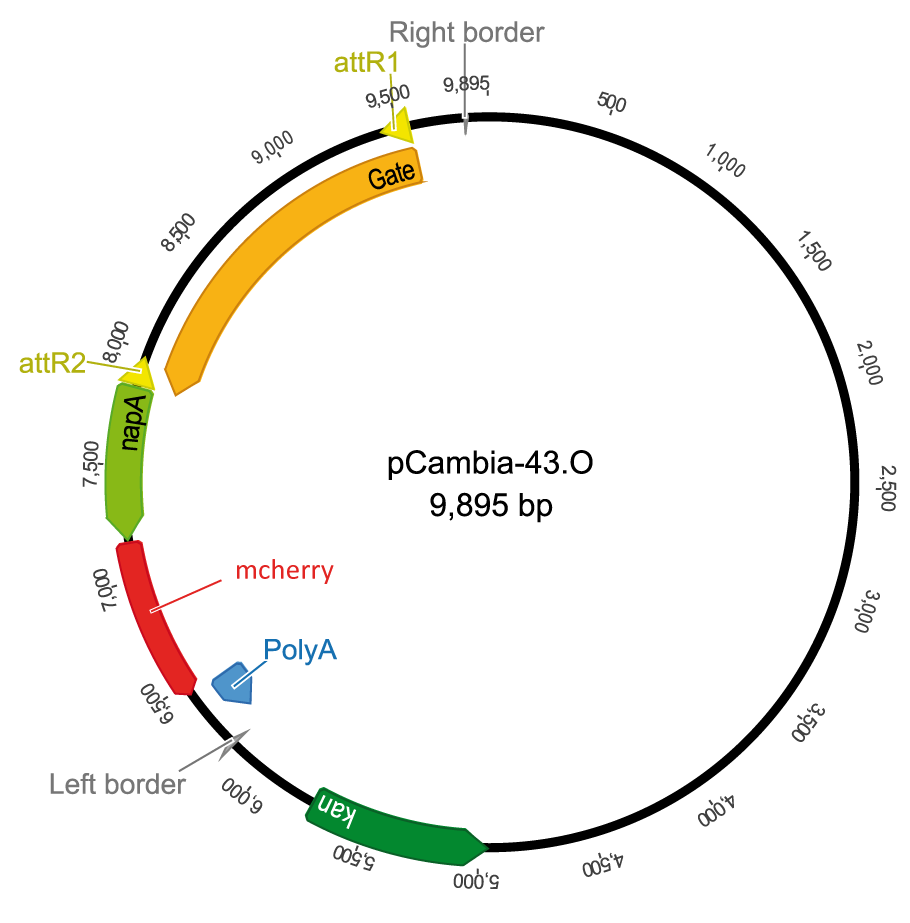


Nucleotide sequence:

gcatgccaaccacagggttcccctcgggatcaaagtactttgatccaacccctccgctgctatagtgcagtcggcttctgacgttcagtgcagccgtcttctgaaaacgacatgtcgcacaagtcctaagttacgcgacaggctgccgccctgcccttttcctggcgttttcttgtcgcgtgttttagtcgcataaagtagaatacttgcgactagaaccggagacattacgccatgaacaagagcgccgccgctggcctgctgggctatgcccgcgtcagcaccgacgaccaggacttgaccaaccaacgggccgaactgcacgcggccggctgcaccaagctgttttccgagaagatcaccggcaccaggcgcgaccgcccggagctggccaggatgcttgaccacctacgccctggcgacgttgtgacagtgaccaggctagaccgcctggcccgcagcacccgcgacctactggacattgccgagcgcatccaggaggccggcgcgggcctgcgtagcctggcagagccgtgggccgacaccaccacgccggccggccgcatggtgttgaccgtgttcgccggcattgccgagttcgagcgttccctaatcatcgaccgcacccggagcgggcgcgaggccgccaaggcccgaggcgtgaagtttggcccccgccctaccctcaccccggcacagatcgcgcacgcccgcgagctgatcgaccaggaaggccgcaccgtgaaagaggcggctgcactgcttggcgtgcatcgctcgaccctgtaccgcgcacttgagcgcagcgaggaagtgacgcccaccgaggccaggcggcgcggtgccttccgtgaggacgcattgaccgaggccgacgccctggcggccgccgagaatgaacgccaagaggaacaagcatgaaaccgcaccaggacggccaggacgaaccgtttttcattaccgaagagatcgaggcggagatgatcgcggccgggtacgtgttcgagccgcccgcgcacgtctcaaccgtgcggctgcatgaaatcctggccggtttgtctgatgccaagctggcggcctggccggccagcttggccgctgaagaaaccgagcgccgccgtctaaaaaggtgatgtgtatttgagtaaaacagcttgcgtcatgcggtcgctgcgtatatgatgcgatgagtaaataaacaaatacgcaaggggaacgcatgaaggttatcgctgtacttaaccagaaaggcgggtcaggcaagacgaccatcgcaacccatctagcccgcgccctgcaactcgccggggccgatgttctgttagtcgattccgatccccagggcagtgcccgcgattgggcggccgtgcgggaagatcaaccgctaaccgttgtcggcatcgaccgcccgacgattgaccgcgacgtgaaggccatcggccggcgcgacttcgtagtgatcgacggagcgccccaggcggcggacttggctgtgtccgcgatcaaggcagccgacttcgtgctgattccggtgcagccaagcccttacgacatatgggccaccgccgacctggtggagctggttaagcagcgcattgaggtcacggatggaaggctacaagcggcctttgtcgtgtcgcgggcgatcaaaggcacgcgcatcggcggtgaggttgccgaggcgctggccgggtacgagctgcccattcttgagtcccgtatcacgcagcgcgtgagctacccaggcactgccgccgccggcacaaccgttcttgaatcagaacccgagggcgacgctgcccgcgaggtccaggcgctggccgctgaaattaaatcaaaactcatttgagttaatgaggtaaagagaaaatgagcaaaagcacaaacacgctaagtgccggccgtccgagcgcacgcagcagcaaggctgcaacgttggccagcctggcagacacgccagccatgaagcgggtcaactttcagttgccggcggaggatcacaccaagctgaagatgtacgcggtacgccaaggcaagaccattaccgagctgctatctgaatacatcgcgcagctaccagagtaaatgagcaaatgaataaatgagtagatgaattttagcggctaaaggaggcggcatggaaaatcaagaacaaccaggcaccgacgccgtggaatgccccatgtgtggaggaacgggcggttggccaggcgtaagcggctgggttgtctgccggccctgcaatggcactggaacccccaagcccgaggaatcggcgtgacggtcgcaaaccatccggcccggtacaaatcggcgcggcgctgggtgatgacctggtggagaagttgaaggccgcgcaggccgcccagcggcaacgcatcgaggcagaagcacgccccggtgaatcgtggcaagcggccgctgatcgaatccgcaaagaatcccggcaaccgccggcagccggtgcgccgtcgattaggaagccgcccaagggcgacgagcaaccagattttttcgttccgatgctctatgacgtgggcacccgcgatagtcgcagcatcatggacgtggccgttttccgtctgtcgaagcgtgaccgacgagctggcgaggtgatccgctacgagcttccagacgggcacgtagaggtttccgcagggccggccggcatggccagtgtgtgggattacgacctggtactgatggcggtttcccatctaaccgaatccatgaaccgataccgggaagggaagggagacaagcccggccgcgtgttccgtccacacgttgcggacgtactcaagttctgccggcgagccgatggcggaaagcagaaagacgacctggtagaaacctgcattcggttaaacaccacgcacgttgccatgcagcgtacgaagaaggccaagaacggccgcctggtgacggtatccgagggtgaagccttgattagccgctacaagatcgtaaagagcgaaaccgggcggccggagtacatcgagatcgagctagctgattggatgtaccgcgagatcacagaaggcaagaacccggacgtgctgacggttcaccccgattactttttgatcgatcccggcatcggccgttttctctaccgcctggcacgccgcgccgcaggcaaggcagaagccagatggttgttcaagacgatctacgaacgcagtggcagcgccggagagttcaagaagttctgtttcaccgtgcgcaagctgatcgggtcaaatgacctgccggagtacgatttgaaggaggaggcggggcaggctggcccgatcctagtcatgcgctaccgcaacctgatcgagggcgaagcatccgccggttcctaatgtacggagcagatgctagggcaaattgccctagcaggggaaaaaggtcgaaaaggtctctttcctgtggatagcacgtacattgggaacccaaagccgtacattgggaaccggaacccgtacattgggaacccaaagccgtacattgggaaccggtcacacatgtaagtgactgatataaaagagaaaaaaggcgatttttccgcctaaaactctttaaaacttattaaaactcttaaaacccgcctggcctgtgcataactgtctggccagcgcacagccgaagagctgcaaaaagcgcctacccttcggtcgctgcgctccctacgccccgccgcttcgcgtcggcctatcgcggccgctggccgctcaaaaatggctggcctacggccaggcaatctaccagggcgcggacaagccgcgccgtcgccactcgaccgccggcgcccacatcaaggcaccctgcctcgcgcgtttcggtgatgacggtgaaaacctctgacacatgcagctcccggagacggtcacagcttgtctgtaagcggatgccgggagcagacaagcccgtcagggcgcgtcagcgggtgttggcgggtgtcggggcgcagccatgacccagtcacgtagcgatagcggagtgtatactggcttaactatgcggcatcagagcagattgtactgagagtgcaccatatgcggtgtgaaataccgcacagatgcgtaaggagaaaataccgcatcaggcgctcttccgcttcctcgctcactgactcgctgcgctcggtcgttcggctgcggcgagcggtatcagctcactcaaaggcggtaatacggttatccacagaatcaggggataacgcaggaaagaacatgtgagcaaaaggccagcaaaaggccaggaaccgtaaaaaggccgcgttgctggcgtttttccataggctccgcccccctgacgagcatcacaaaaatcgacgctcaagtcagaggtggcgaaacccgacaggactataaagataccaggcgtttccccctggaagctccctcgtgcgctctcctgttccgaccctgccgcttaccggatacctgtccgcctttctcccttcgggaagcgtggcgctttctcatagctcacgctgtaggtatctcagttcggtgtaggtcgttcgctccaagctgggctgtgtgcacgaaccccccgttcagcccgaccgctgcgccttatccggtaactatcgtcttgagtccaacccggtaagacacgacttatcgccactggcagcagccactggtaacaggattagcagagcgaggtatgtaggcggtgctacagagttcttgaagtggtggcctaactacggctacactagaaggacagtatttggtatctgcgctctgctgaagccagttaccttcggaaaaagagttggtagctcttgatccggcaaacaaaccaccgctggtagcggtggtttttttgtttgcaagcagcagattacgcgcagaaaaaaaggatctcaagaagatcctttgatcttttctacggggtctgacgctcagtggaacgaaaactcacgttaagggattttggtcatgcattctaggtactaaaacaattcatccagtaaaatataatattttattttctcccaatcaggcttgatccccagtaagtcaaaaaatagctcgacatactgttcttccccgatatcctccctgatcgaccggacgcagaaggcaatgtcataccacttgtccgccctgccgcttctcccaagatcaataaagccacttactttgccatctttcacaaagatgttgctgtctcccaggtcgccgtgggaaaagacaagttcctcttcgggcttttccgtctttaaaaaatcatacagctcgcgcggatctttaaatggagtgtcttcttcccagttttcgcaatccacatcggccagatcgttattcagtaagtaatccaattcggctaagcggctgtctaagctattcgtatagggacaatccgatatgtcgatggagtgaaagagcctgatgcactccgcatacagctcgataatcttttcagggctttgttcatcttcatactcttccgagcaaaggacgccatcggcctcactcatgagcagattgctccagccatcatgccgttcaaagtgcaggacctttggaacaggcagctttccttccagccatagcatcatgtccttttcccgttccacatcataggtggtccctttataccggctgtccgtcatttttaaatataggttttcattttctcccaccagcttatataccttagcaggagacattccttccgtatcttttacgcagcggtatttttcgatcagttttttcaattccggtgatattctcattttagccatttattatttccttcctcttttctacagtatttaaagataccccaagaagctaattataacaagacgaactccaattcactgttccttgcattctaaaaccttaaataccagaaaacagctttttcaaagttgttttcaaagttggcgtataacatagtatcgacggagccgattttgaaaccgcggtgatcacaggcagcaacgctctgtcatcgttacaatcaacatgctaccctccgcgagatcatccgtgtttcaaacccggcagcttagttgccgttcttccgaatagcatcggtaacatgagcaaagtctgccgccttacaacggctctcccgctgacgccgtcccggactgatgggctgcctgtatcgagtggtgattttgtgccgagctgccggtcggggagctgttggctggctggtggcaggatatattgtggtgtaaacaaattgacgcttagacaacttaataacacattgcggacgtttttaatgtactgaattaacgccgaattaattcgggggatctggattttagtactggattttggttttaggaattagaaattttattgatagaagtattttacaaatacaaatacatactaagggtttcttatatgctcaacacatgagcgaaaccctataggaaccctaattcccttatctgggaactactcacacattattatggagaaactcgagttacttgtacagctcgtccatgccgccggtggagtggcggccctcggcgcgttcgtactgttccacgatggtgtagtcctcgttgtgggaggtgatgtccaacttgatgttgacgttgtaggcgccgggcagctgcacgggcttcttggccttgtaggtggtcttgacctcagcgtcgtagtggccgccgtccttcagcttcagcctctgcttgatctcgcccttcagggcgccgtcctcggggtacatccgctcggaggaggcctcccagcccatggtcttcttctgcattacggggccgtcggaggggaagttggtgccgcgcagcttcaccttgtagatgaactcgccgtcctgcagggaggagtcctgggtcacggtcaccacgccgccgtcctcgaagttcatcacgcgctcccacttgaagccctcggggaaggacagcttcaagtagtcggggatgtcggcggggtgcttcacgtaggccttggagccgtacatgaactgaggggacaggatgtcccaggcgaagggcagggggccacccttggtcaccttcagcttggcggtctgggtgccctcgtaggggcggccctcgccctcgccctcgatctcgaactcgtggccgttcacggagccctccatgtgcaccttgaagcgcatgaactccttgatgatggccatgttatcctcctcgcccttgctcaccatgtcgactgtttttaatcttgtttgtattgatgagttttggtttgagtaaagagtgaagccgatgagttaatttataggctataaaggagatttgcatggcgatcacgtgtaataatgcatgcacgcatgtgattgtatgtgtgtgctgtgagagagaagctcttaggtgtttgaagggagtgacaagtggcgaagaaaaacaattctccgcggctgcatgctatgtgtaacgtgtagctaatgttctggcatggcatcttatgaacgattctttttaaaaacaaggtaaaaacttaacttcataaaattaaaaaaaaaacgtttactaagttggtttaaaaggggatgagagtctataaattttggaggtagtgccgttgggaatataaattgggagcttaatcagaattatagaagttaaagttgatttagtcacggtcaatataaattgggaatttgagtcaaaatcttccaaattcggaatccgtcttgttacacccggtggataggagccgaacggtttgaaaatacttgaaatgtggatgcaggtgcaggctggtttaattttatgttgaatggatacatgtcaatcgaatttgagttataggtacacattttactctgatactaaaatgtaacatttgtctcaagaatgggtaggtcatccttgagctcggtaccctcaaccactttgtacaagaaagctgaacgagaaacgtaaaatgatataaatatcaatatattaaattagattttgcataaaaaacagactacataatactgtaaaacacaacatatccagtcactatggtcgacctgcagactggctgtgtataagggagcctgacatttatattccccagaacatcaggttaatggcgtttttgatgtcattttcgcggtggctgagatcagccacttcttccccgataacggagaccggcacactggccatatcggtggtcatcatgcgccagctttcatccccgatatgcaccaccgggtaaagttcacgggagactttatctgacagcagacgtgcactggccagggggatcaccatccgtcgcccgggcgtgtcaataatatcactctgtacatccacaaacagacgataacggctctctcttttataggtgtaaaccttaaactgcatttcaccagtccctgttctcgtcagcaaaagagccgttcatttcaataaaccgggcgacctcagccatcccttcctgattttccgctttccagcgttcggcacgcagacgacgggcttcattctgcatggttgtgcttaccagaccggagatattgacatcatatatgccttgagcaactgatagctgtcgctgtcaactgtcactgtaatacgctgcttcatagcacacctctttttgacatacttcgggtatacatatcagtatatattcttataccgcaaaaatcagcgcgcaaatacgcatactgttatctggcttttagtaagccggatccagatctttacgccccgccctgccactcatcgcagtactgttgtaattcattaagcattctgccgacatggaagccatcacagacggcatgatgaacctgaatcgccagcggcatcagcaccttgtcgccttgcgtataatatttgcccatggtgaaaacgggggcgaagaagttgtccatattggccacgtttaaatcaaaactggtgaaactcacccagggattggctgagacgaaaaacatattctcaataaaccctttagggaaataggccaggttttcaccgtaacacgccacatcttgcgaatatatgtgtagaaactgccggaaatcgtcgtggtattcactccagagcgatgaaaacgtttcagtttgctcatggaaaacggtgtaacaagggtgaacactatcccatatcaccagctcaccgtctttcattgccatacggaattccggatgagcattcatcaggcgggcaagaatgtgaataaaggccggataaaacttgtgcttatttttctttacggtctttaaaaaggccgtaatatccagctgaacggtctggttataggtacattgagcaactgactgaaatgcctcaaaatgttctttacgatgccattgggatatatcaacggtggtatatccagtgatttttttctccattttagcttccttagctcctgaaaatctcgccggatcctaactcaaaatccacacattatacgagccggaagcataaagtgtaaagcctggggtgcctaatgcggccgccatagtgactggatatgttgtgttttacagtattatgtagtctgttttttatgcaaaatctaatttaatatattgatatttatatcattttacgtttctcgttcagcttttttgtacaaacttgttgatccctcttggcactggccgtcgttttacaacgtcgtgactgggaaaaccctggcgttacccaacttaatcgccttgcagcacatccccctttcgccagctggcgtaatagcgaagaggcccgcaccgatcgcccttcccaacagttgcgcagcctgaatggcgaatgctagagcagcttgagcttggatcagattgtcgtttcccgccttcagtttaaactatcagtgtttgacaggatatattggcgggtaaacctaagagaaaagagcgtttattagaataacggatatttaaaagggcgtgaaaaggtttatccgttcgtccatttgtatgt
